# Proteomics Studies on the three Larval Stages of Development and Metamorphosis of *Babylonia areolata*

**DOI:** 10.1038/s41598-018-24645-z

**Published:** 2018-04-19

**Authors:** Minghui Shen, Guilan Di, Min Li, Jingqiang Fu, Qi Dai, Xiulian Miao, Miaoqin Huang, Weiwei You, Caihuan Ke

**Affiliations:** 1 State Key Laboratory of Marine Environmental Science, College of Ocean and Earth Sciences, Xiamen University, Xiamen, 361005 China; 2 College of Fisheries, Henan Normal University, Xinxiang, 453007 China; 3 Hainan Academy of Ocean and Fisheries Sciences, Haikou, 570206 China; 4 College of Life Sciences, Liaocheng University, Liaocheng, 252059 China

## Abstract

The ivory shell, *Babylonia areolata*, is a commercially important aquaculture species in the southeast coast of mainland China. The middle veliger stage, later veliger stage, and juvenile stage are distinct larval stages in *B*. *areolata* development. In this study, we used label-free quantification proteomics analysis of the three developmental stages of *B*. *areolata*. We identified a total of 5,583 proteins, of which 1,419 proteins expression level showed significant differential expression. The results of gene ontology enrichment analysis showed that the number of proteins involved in metabolic and cellular processes were the most abundant. Those proteins mostly had functions such as binding, catalytic activity and transporter activity. The results of Kyoto Encyclopedia of Genes and Genomes enrichment analysis showed that the number of proteins involved in the ribosome, carbon metabolism, and lysosome pathways were the most abundant, indicating that protein synthesis and the immune response were active during the three stages of development. This is the first study to use proteomics and real-time PCR to study the early developmental stages of *B*. *areolata*, which could provide relevant data on gastropod development. Our results provide insights into the novel aspects of protein function in shell formation, body torsion, changes in feeding habits, attachment and metamorphosis, immune-related activities in *B*. *areolata* larvae.

## Introduction

Embryonic and larval development is an important phylogenetic event^1^, and animal embryology research contributes to development of the aquaculture industry and environmental pollution monitoring^2^. Some gastropod larval development includes a pelagic phase (a period of free living) and benthic phase. Larvae undergo metamorphosis, transitioning to benthonic postlarva or juveniles^3^. Complex morphological and physiological changes occur during the transition between these stages, and morphological differences in the larvae development of gastropods have been reported^4–6^. Larval settlement and metamorphosis is a crucial transition period associated with the evolution of metazoans, as well as differentiation and speciation^7^. The attachment and metamorphosis of pelagic gastropod larvae directly affects the population distribution, quantity change, and species evolution^8,9^. Even in cultured gastropods, metamorphosis is a vital step for the aquaculture facility, because gastropod animals that have successfully settled usually show increased survival.

The embryonic development of gastropods includes several unique features including shell formation, head–foot differentiation of the changes of body shape, deposition of minerals, and pigment deposition in a protein matrix^10^. Studies about the gene expression of mollusk larvae during development have mainly been focused on anatomical structural ontogenesis^11,12^; there are molecular studies related metamorphosis for commercially aquacultured species like bivalve oysters^13^. However, the molecular mechanisms underlying of gastropod metamorphosis is still fragmentary.

Species of the genus *Babylonia* (Mollusca, Gastropoda, Neogastropoda, Babyloniidae) have been the most promising economic marine gastropod in this century, because of its wide distribution along most parts of Asian coastlines, delicate flavor, and high market reception^14,15^. *Babylonia areolata* are common species in China^16^, and are good study materials for both scientific studies on the early development and metamorphosis of gastropods and the artificial breeding technology of the genus *Babylonia*. Huang *et al*.^17^ observed significant differences in the developmental events of the *B*. *areolata*, including cleavage, formation of a cap-shaped blastoderm, the blastula stage, and embryo begins to turn around slowly. The early veliger stage appears 60–63 h after spawning, which is distinguished by the appearance of the velum, feet, and shell. The middle veliger stage includes the veliger in the egg sac and the planktonic juvenile, early stage still contains the yolk sac. Then the larva crawls out to form the egg sac and leads a pelagic life for 1–2 d, after which the yolk sac disappears completely, and the veliger enters the late veliger stage with a more rapid heartbeat and larger and elongated foot. After 10–12 d at 27. 5–28. 5 °C, the late veliger stage sinks to the substratum and leads a benthic life, the velum gradually degenerates and atrophies, and the veliger completes its metamorphosis and enters the juvenile snail stage, which is marked by the ability to creeping and the appearance of brown stripes on the shell^17^.

Genomic and transcriptomic analyses have been used to study larval development, and the transcriptome of the early development stages of the California Sea Hare *Aplysia californica* (a marine opisthobranch gastropod mollusk) has been reported^18^. However, current genomic data are insufficient to reveal the molecular mechanisms underlying the complex processes of embryonic development^19^. Proteins determine phenotypes, which can be considered snapshots of genome expression^20^. There has been great interest in studying reproduction-related proteins; such studies have the potential to address questions on speciation and evolution^21,22^. Phenotypes are more complex than genomic expression, due to lack of a direct correlation between gene expression intensity and protein abundance; thus, proteomic studies are powerful tools for identifying novel proteins involved in developmental processes. For example, proteomics have been used to identify novel proteins from the calcifying shell matrix of the manila clam *Venerupis philippinarum*^23^, pacific oyster *Crassostrea gigas*^24^, mollusc shell proteins in the *Haliotis tuberculata*^25^, and scallop attachment^26^. Proteomic profiling has also been applied to embryo studies of several molluscs including the golden apple snail (*Pomacea canaliculata*)^27^, abalone reproduction^28^, and the Pacific oyster (*C*. *gigas*)^19^. Proteomic analysis is a crucial approach for studies on animal metamorphosis^13^. Proteomic has been applied in many marine invertebrates, for example, polychaetes *Pseudopolydora vexillosa*^29^, *Bugula neritina*^30^, barnacles^31,32^, bivalve oysters^13^ and small abalone *Haliotis diversicolor*^33^. These studies provide comprehensive information on the underlying molecular mechanisms, and identified proteins that may be related to metamorphosis. Proteomic studies on *B*. *areolata* embryos have not been previously reported, for the studies on gastropod *B*. *areolata* metamorphosis, a proteomic analysis would complement and expand current knowledge.

Label-free quantification proteomics is facilitated by quantitative mass spectrometry, and this technique involves the application of a label-free quantification algorithm used in Maxquant for proteomics data. To thoroughly evaluate larval proteome changes, we investigated the differential protein abundance during the three development stages of *B*. *areolata* using a label-free quantification proteomic approach. Data were further examined with Perseus, and annotated to gene ontology (GO) terms and Kyoto Encyclopedia of Genes and Genomes (KEGG) pathways by evaluating the GO terms, KEGG pathway enrichment, and mRNA expression of differentially expressed proteins (DEPs) to assess the shell formation, body torsion, changes in feeding habits, and metamorphosis of *B*. *areolata* larvae through proteomics analysis, and to explore the larval development and metamorphosis mechanisms based on proteomics. This study could provide reference data for related studies of other marine gastropods from a proteomics perspective.

## Results

### Three Stages of Larval Development

**Figure 1:**
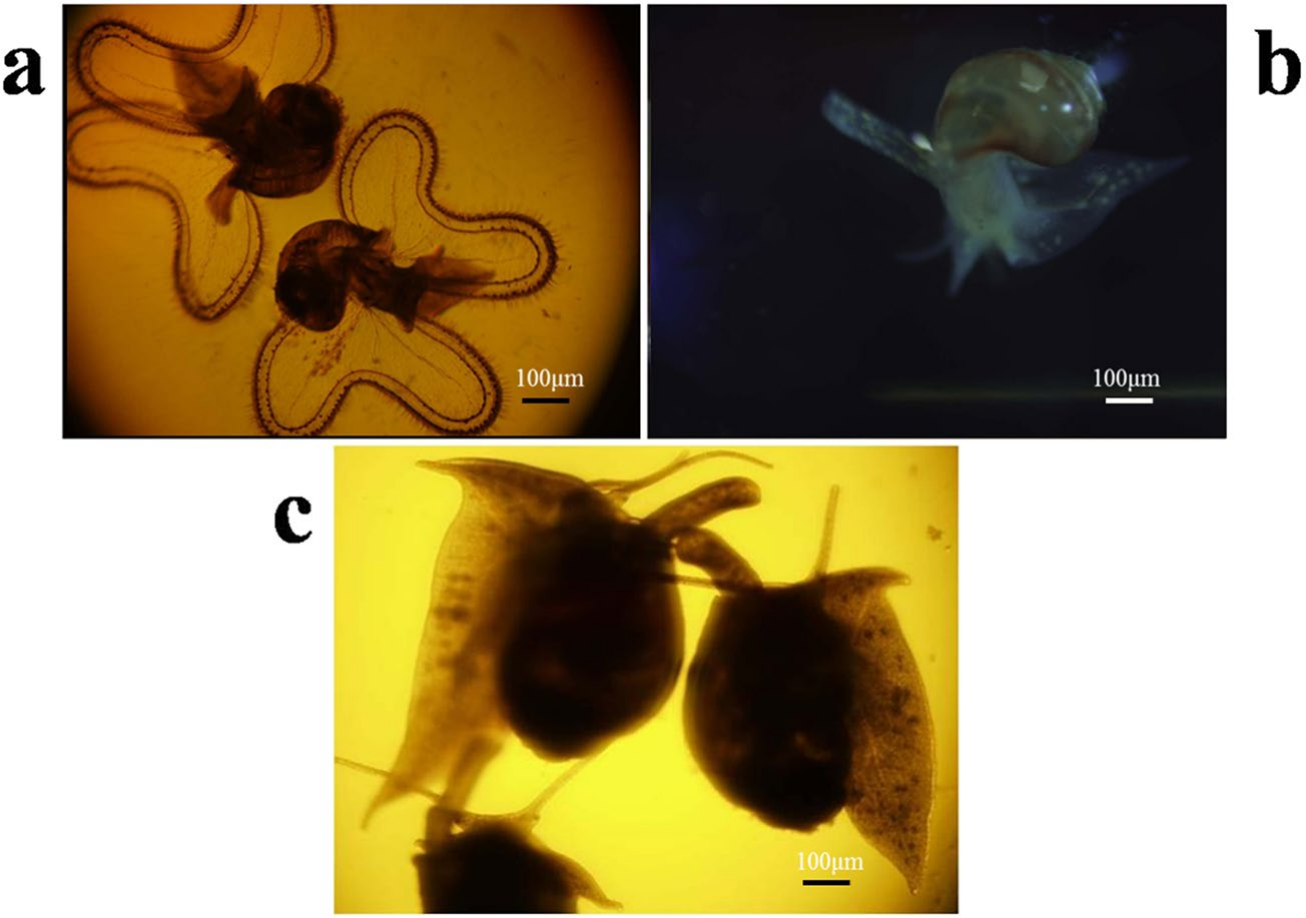
Embryonic development of *Babylonia areolata*. (**a**) Veliger before attachment (**b**) Veliger at the later metamorphosis stage (velum atrophy) (**c**) Juvenile *B*. *areolata*, the feeding habits change from phytophagy to sarcophagy.

Three groups of larval *B*. *areolata* at different developmental stages including the middle veliger stage before attachment (ZRZ-III), later veliger stage (velum atrophy) (ZRZ-V), and juvenile stage (ZRZ-VI) were used. Each group was replicated three times. The three stages of larval development are shown in Fig. 1a–c. The early veliger stage is distinguished by the appearance of the velum, feet, and shell. The middle veliger stage is a pelagic life for 1–2 d and the average shell height of the middle veliger larvae is about 520 μm, and the late veliger stage is characterized by a more rapid heartbeat and larger and elongated foot (Fig. 1a). After 10–12 d, the late veliger stage sinks to the substratum and leads a benthic life, the velum gradually degenerates and atrophies, after which the veliger completes its metamorphosis (Fig. 1b) and enters the juvenile snail stage (Fig. 1c).

### Sodium dodecyl sulfate polyacrylamide gel electrophoresis of the extracted proteins

**Figure 2:**
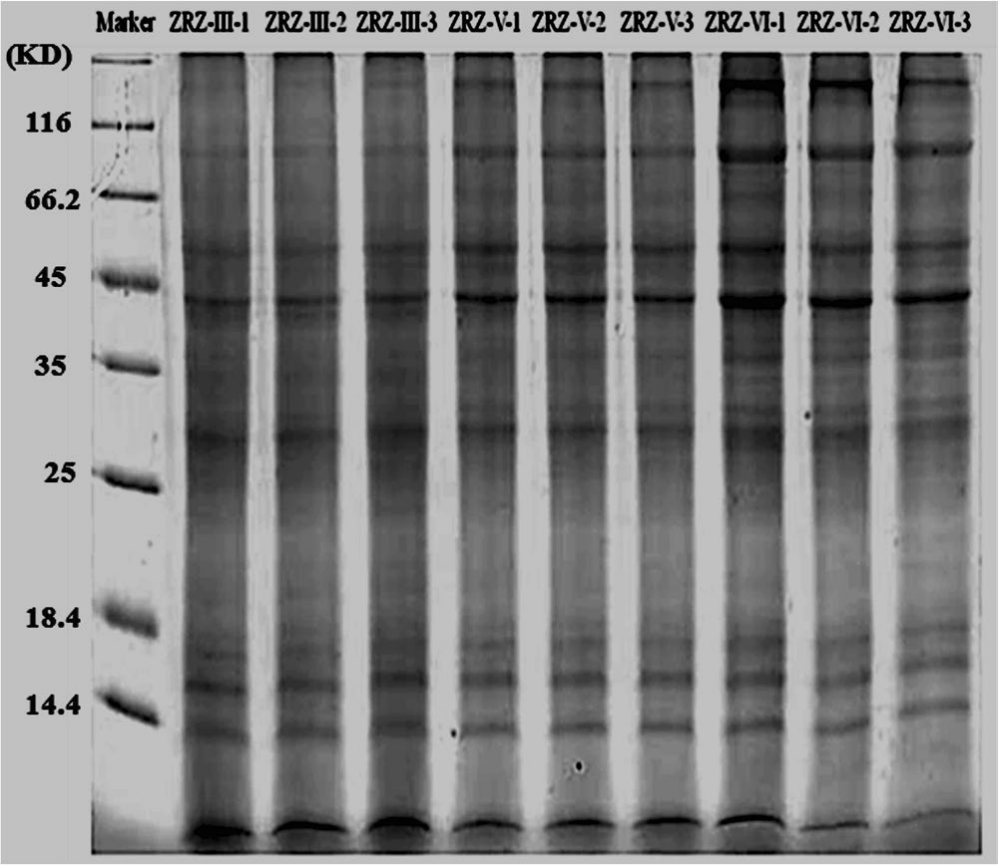
SDS-PAGE analysis. Marker show the protein molecular weight standard, ZRZ-III show the middle veliger stage, ZRZ-V represent later veliger stage, and ZRZ-VI represent juvenile snail stage.

The determined protein concentration of the sample is shown in Table 1. The extracted proteins were analyzed by sodium dodecyl sulfate polyacrylamide gel electrophoresis (SDS-PAGE). The samples at the three developmental stages showed clear protein bands (Fig. 2), three parallel experiments were performed with good repeatability for each developmental stage.

**Table 1:** Protein and peptide fragment concentration determination.

Sample ID	Concentration (μg/μL)	Volume (μL)	Total Quantity (μg)	Type Evaluation	Peptide Concentration (μg/μL)
ZRZ_III-1	26.83	200	5,400	A	1.84
ZRZ_III-2	26.75	200	5,400	A	1.73
ZRZ_III-3	26.26	200	5,400	A	1.68
ZRZ_V-1	24.11	200	4,800	A	1.59
ZRZ_V-2	24.35	200	4,800	A	1.75
ZRZ_V-3	24.28	200	4,800	A	1.61
ZRZ_VI-1	15.05	200	3,000	A	1.83
ZRZ_VI-2	10.20	200	2,000	A	1.01
ZRZ_VI-3	4.26	200	850	A	1.63

### Analysis of the Significant Differences in Protein Abundance

All of the raw liquid chromatography-tandem mass spectrometry (LC-MS/MS) data from the three developmental stages were analyzed by one-way analysis of variance (ANOVA). Subsequently, the DEPs (p < 0.05) were selected. From the raw data, a total of 5,583 proteins, including 1,419 DEPs that satisfied the screening criteria, were present (ratio > ±2 and p < 0.05). A total of 721 protein groups were identified and 1,930 specific polypeptides were identified. The quantitative protein sequence information was extracted in batch from the self-built library. The resulting protein sequences were analyzed by BLAST, thereby obtaining a total of 1132 highly similar proteins. These proteins were ranked by similarity, and the top 28 proteins are shown in Table 2. The pairwise comparison of the three different developmental stages is presented in Table 3. The number of DEPs between ZRZ_VI and ZRZ_III was large, and 733 proteins were qualitatively different. Among the proteins with varying expression levels, 281 and 259 proteins were upregulated and downregulated, respectively, at ZRZ_VI. The differentially expressed proteins with important physiological functions between ZRZ_V and ZRZ_III, ZRZ_VI and ZRZ_III, and ZRZ_VI and ZRZ_V with protein volumes calculated are showed in Supplementary Tables 1–3.

**Table 2:** Top BLAST hits.

Sequence Name	Hit Description	Bit Score	Sequences (n)
c268231_g4	spectrin beta chain-like isoform X3	3749.9	1986
c268656_g2	myosin heavy chain, striated muscle-like isoform X11	2845.07	1599
c268768_g1	dynein beta chain, ciliary-like	3054.23	1450
c265341_g2	rootletin-like isoform X6	2313.11	1390
c267248_g4	filamin-A-like isoform X4	2758.79	1383
c260248_g1	dynein heavy chain 5, axonemal-like	2678.66	1307
c268675_g1	hypothetical protein LOTGIDRAFT_218488	2144.01	1165
c267808_g1	splicing factor 3B subunit 1-like isoform X1	2052.33	1054
c266291_g1	spectrin alpha chain-like isoform X7	2029.22	1025
c268690_g2	twitchin-like	2174.05	1022
c268077_g2	uncharacterized protein LOC101849429	2081.6	1008
c261417_g3	dynein heavy chain 10, axonemal-like	1841.63	1004
c268754_g1	uncharacterized protein LOC105329380 isoform X2	1453.73	993
c268643_g1	deleted in malignant brain tumors 1 protein-like isoform X2	1802.72	976
c268439_g1	hypothetical protein LOTGIDRAFT_218145	1899.02	968
c267364_g1	translational activator GCN1	1925.98	946
c268713_g4	dynein beta chain, ciliary-like	1924.83	937
c268768_g2	dynein beta chain, ciliary-like	1825.83	937
c263230_g7	dynein heavy chain 7, axonemal-like	1858.57	919
c268195_g2	sodium/potassium-transporting ATPase subunit alpha-like	1798.1	894
c264969_g2	calcium-transporting ATPase sarcoplasmic/endoplasmic reticulum type-like	1763.04	865
c258154_g7	hypothetical protein LOTGIDRAFT_137237	1788.08	839
c265761_g2	uncharacterized protein LOC101848762	1650.18	835
c268389_g3	laminin subunit beta-1-like	1594.71	831
c256031_g1	dynein heavy chain 1, axonemal-like	1699.49	823
c268690_g3	twitchin-like	1499.57	811
c260032_g1	twitchin-like	1611.66	808
c267456_g1	armadillo repeat-containing protein 4-like	1445.64	789
c264438_g1	spectrin alpha chain-like isoform X7	1513.82	778
c267460_g1	ghypothetical protein LOTGIDRAFT_233044	1642.48	777
c258721_g2	vigilin-like	1579.69	773

**Table 3:** The number of differential proteins.

	V/III		VI/III		VI/V	
	Qualitative difference	Quantitative difference	Qualitative difference	Quantitative difference	Qualitative difference	Quantitative difference
Up-regulation	214	74	348	281	213	168
Down-regulation	90	62	385	259	368	206
The number of proteins	304	3657	733	3253	581	3461

### Cluster Analysis

**Figure 3:**
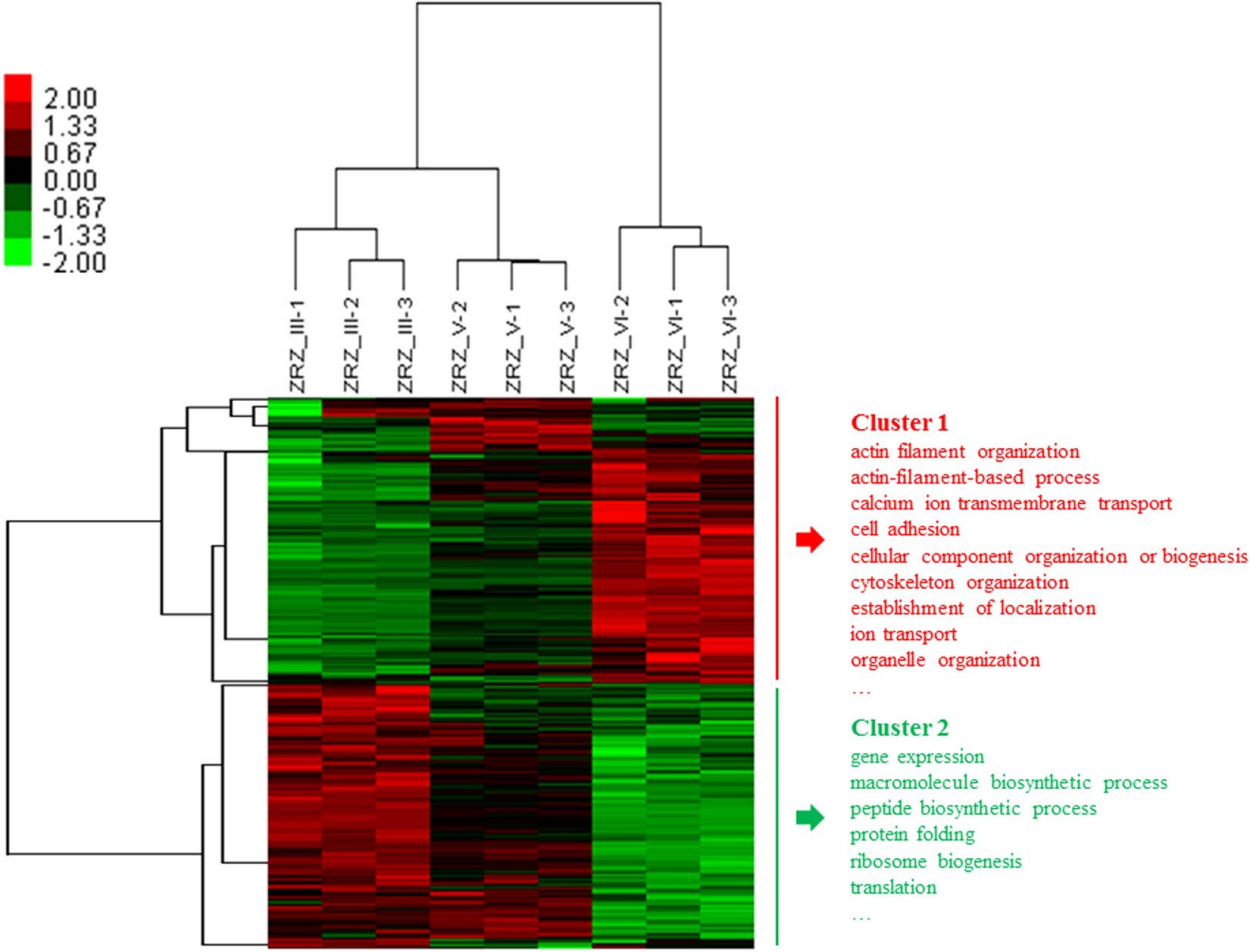
Cluster analysis of DEPs. Heat map showed the clustered data, each colored cell represents a protein abundance value. Colors ranging from green to red represent protein abundance from the highest level of down-regulation to the highest level of up-regulation, respectively.

Cluster analysis clusters and categorizes data on the basis of similarity. The cluster grouping results showed that in terms of mode similarity, the intra-group data were relatively high and the inter-group data were low. The protein cluster analysis results of the larval *B*. *areolata* at the three developmental stages are shown in Fig. 3. In the horizontal direction, data on the three stages were divided into three branches; ZRZ_III and ZRZ_V, which were of the same branch; and ZRZ_VI, which was of another branch. In the vertical direction, the DEPs were divided into two large branches. The protein expression of the first branch was gradually upregulated with varying developmental stage and mainly involved in cell adhesion, cytoskeleton construction, ion transport, and other processes. The second branch was downregulated and mainly involved in gene expression, protein synthesis, and modification.

### GO Functional Annotations

**Figure 4:**
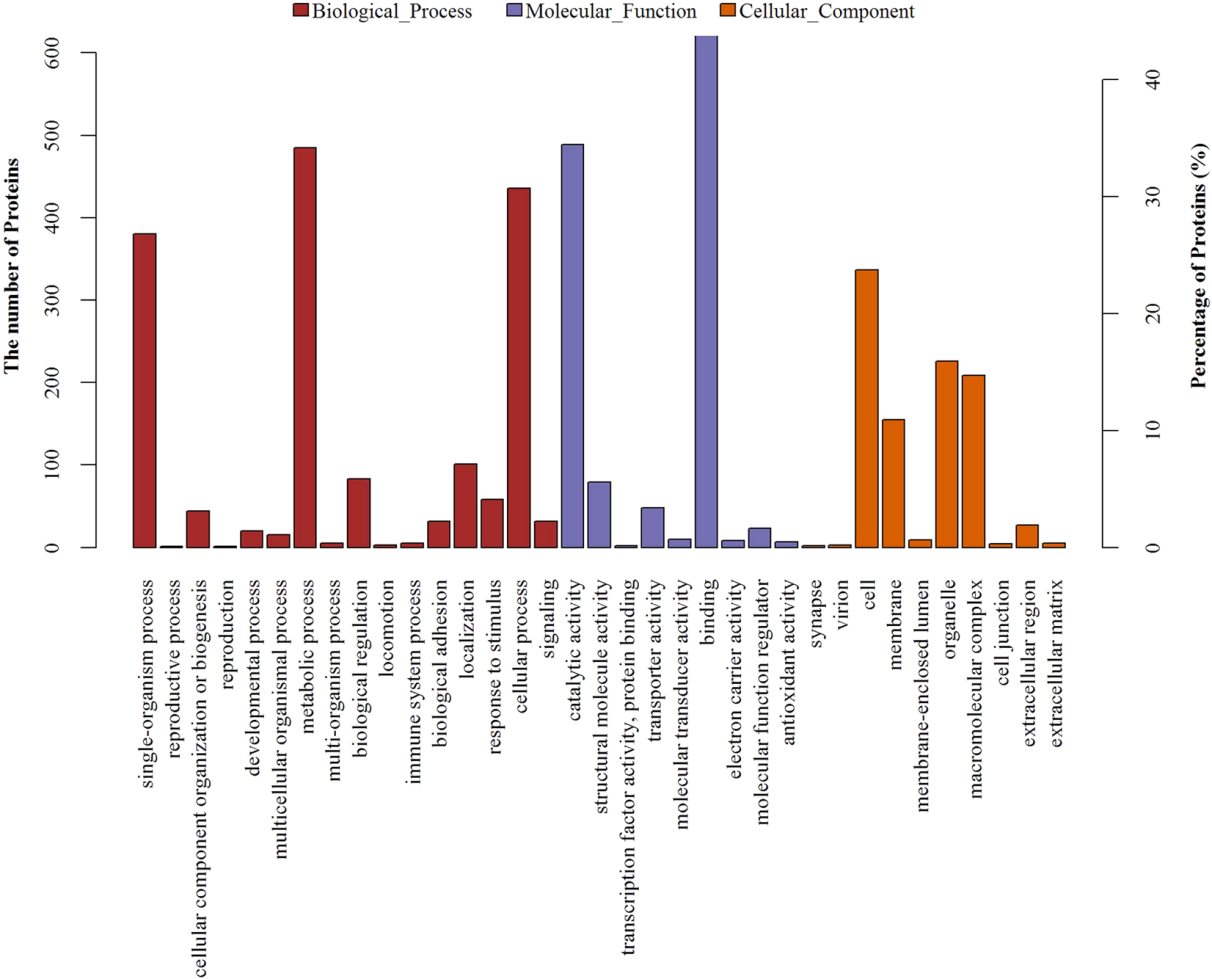
GO Functional classification.

GO describes genes in the organism and the attributes of gene products from three aspects, namely biological processes (BPs), molecular functions (MFs), and cellular component (CCs). The identified proteins were analyzed by GO annotations in three larval stages, the GO functional classification is shown in Fig. 4. Using the BP term, the number of proteins participating in metabolism and those involved in cellular and single organism processes was the first and second largest groups, respectively. Using the MF term, the number of proteins involved in binding and related to catalytic activity was the first and second largest groups, respectively. Using the CC term, the number of proteins involved in cells was the first largest, followed by those related to organelles, the macromolecular complex, and the membrane, respectively. Proteins with high similarity were annotated with GO terms, and the MF results are shown in Table 4. The results showed that most of these DEPs exhibited binding functions.

**Table 4:** GO molecular fuction annotation of Top BLAST hits.

Sequence Name	Hit Description	GO:MF
c268231_g4	spectrin beta chain-like isoform X3	actin binding
c268656_g2	myosin heavy chain, striated muscle-like isoform X11	ATP binding
c268768_g1	dynein beta chain, ciliary-like	ATP binding
c265341_g2	rootletin-like isoform X6	centrosome organization
c267248_g4	filamin-A-like isoform X4	actin filament binding
c260248_g1	dynein heavy chain 5, axonemal-like	calcium ion binding
c268675_g1	hypothetical protein LOTGIDRAFT_218488	calcium ion binding
c267808_g1	splicing factor 3B subunit 1-like isoform X1	nucleotide binding
c266291_g1	spectrin alpha chain-like isoform X7	phospholipid binding
c268690_g2	twitchin-like	nucleotide binding
c268077_g2	uncharacterized protein LOC101849429	lipid transporter activity
c261417_g3	dynein heavy chain 10, axonemal-like	ATPase activity
c268754_g1	uncharacterized protein LOC105329380 isoform X2	zinc ion binding
c268643_g1	deleted in malignant brain tumors 1 protein-like isoform X2	scavenger receptor activi
c268439_g1	hypothetical protein LOTGIDRAFT_218145	calcium ion binding
c267364_g1	translational activator GCN1	kinase regulator activity
c268713_g4	dynein beta chain, ciliary-like	ATP binding
c268768_g2	dynein beta chain, ciliary-like	ATP binding
c263230_g7	dynein heavy chain 7, axonemal-like	ATPase activity
c268195_g2	sodium/potassium-transporting ATPase subunit alpha-like	metal ion binding
c264969_g2	calcium-transporting ATPase sarcoplasmic/endoplasmic reticulum type-like	calcium-transporting ATPase activity
c258154_g7	hypothetical protein LOTGIDRAFT_137237	calcium ion binding
c265761_g2	uncharacterized protein LOC101848762	hydrolase activity
c268389_g3	laminin subunit beta-1-like	protein binding
c256031_g1	dynein heavy chain 1, axonemal-like	microtubule-based movement
c268690_g3	twitchin-like	nucleotide binding
c260032_g1	twitchin-like	protein kinase activity
c267456_g1	armadillo repeat-containing protein 4-like	protein binding
c264438_g1	spectrin alpha chain-like isoform X7	calcium ion binding
c267460_g1	hypothetical protein LOTGIDRAFT_233044	calcium ion binding
c258721_g2	vigilin-like	RNA binding
c265676_g3	collagen alpha-3(VI) chain-like isoform X9	protein binding
c264079_g1	cilia- and flagella-associated protein 58-like	protein binding

### GO Enrichment Analysis of DEPs

**Figure 5:**
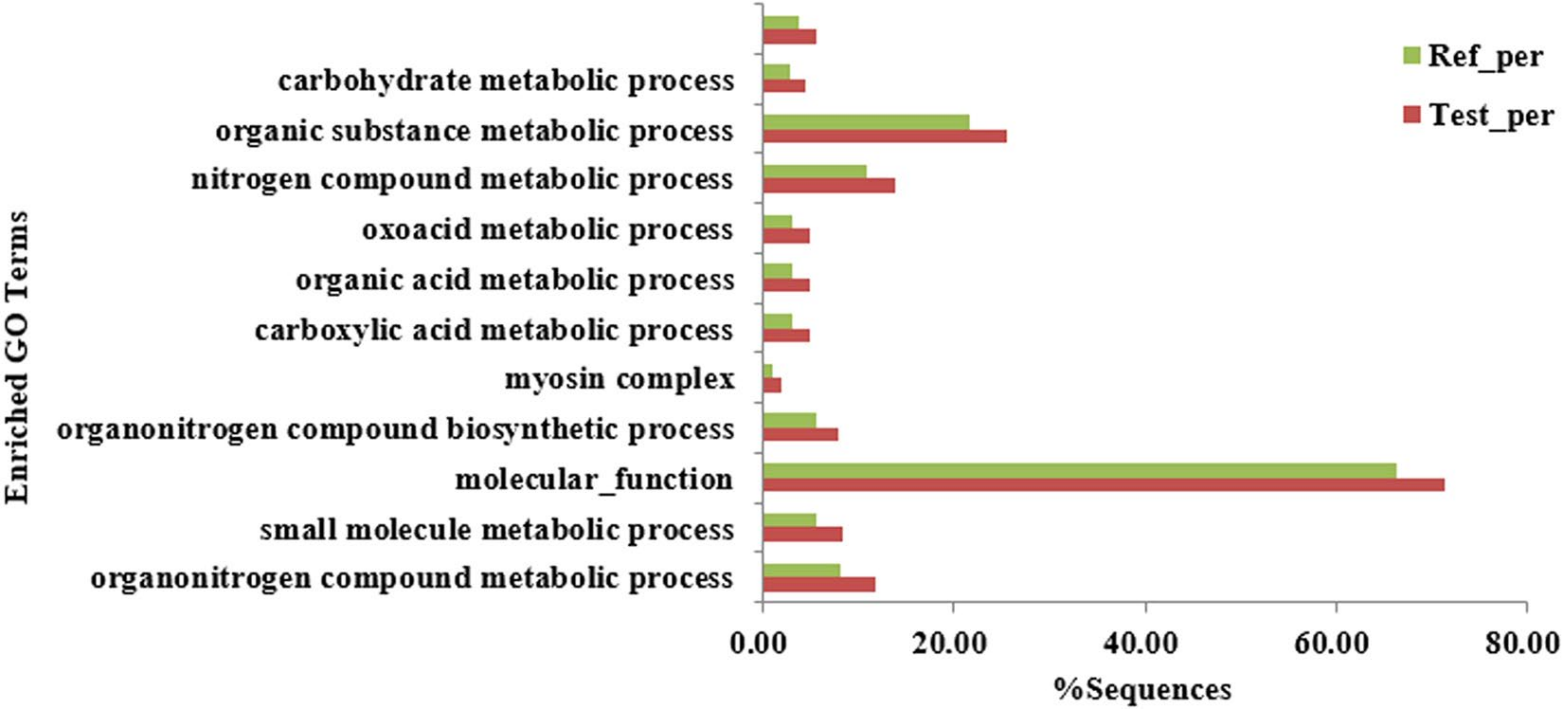
Significantly enriched GO terms. Ref_per: Reference set, refers to the proportion of a GO classification in a total of identified proteins; Test_per: Test set; refers to the proportion of a GO classification in the differential identified proteins. This also applies to the following figures.

The GO annotation of the target protein set allowed the classification of these proteins according to BP, MF, and CC. The proportion of proteins in each class may indicate the effects of each of the three different developmental stages on each GO class. The differentially expressed proteins were analyzed by GO annotations, statistical analysis of the significantly enriched GO terms from data of the three developmental stages is shown in Fig. 5. The results showed that the most significantly enriched GO term was MF, organic substance metabolic process, and nitrogen compound metabolic process, in that order.

### KEGG Pathway Enrichment Analysis of DEPs

**Figure 6:**
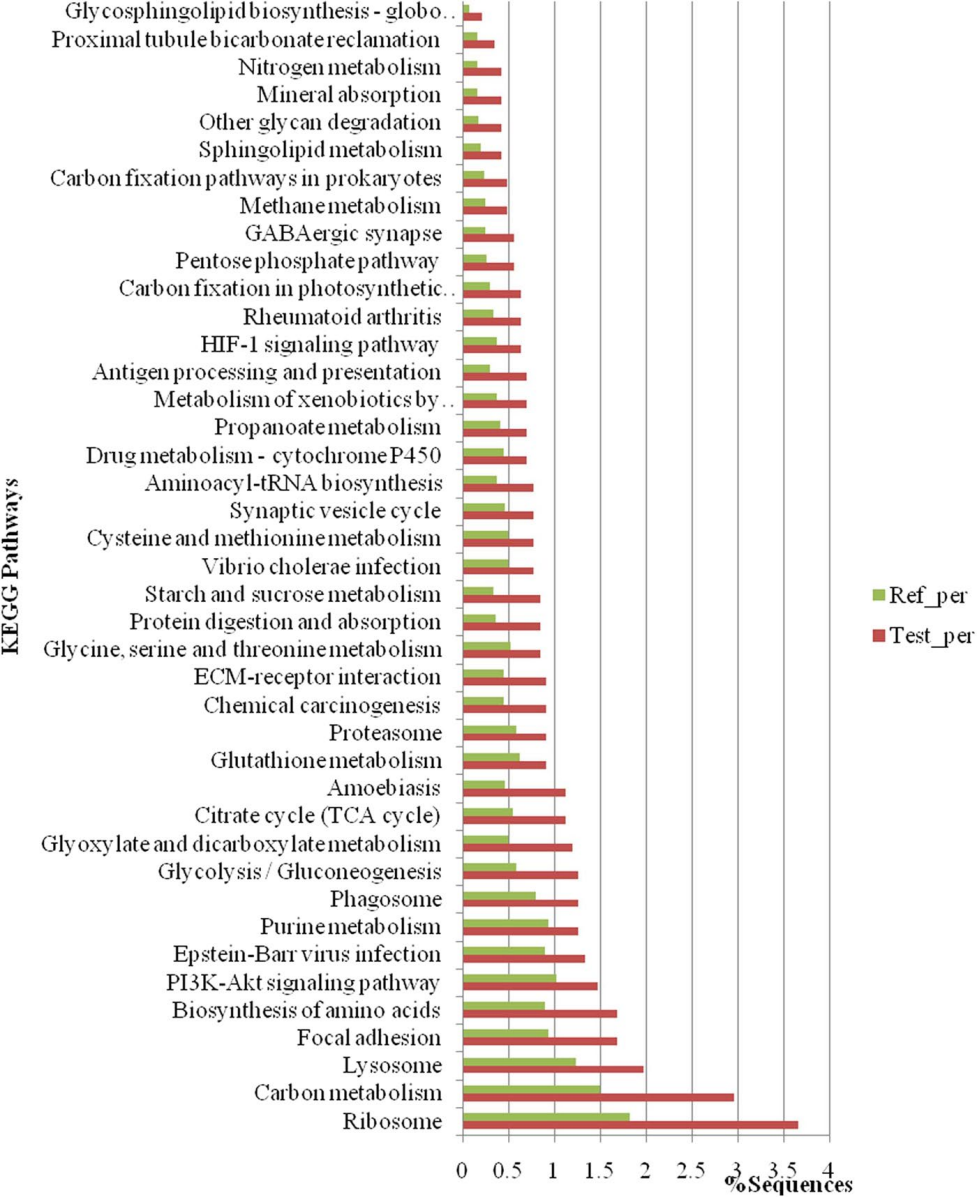
Significantly enriched KEGG pathways.

**Figure 7:**
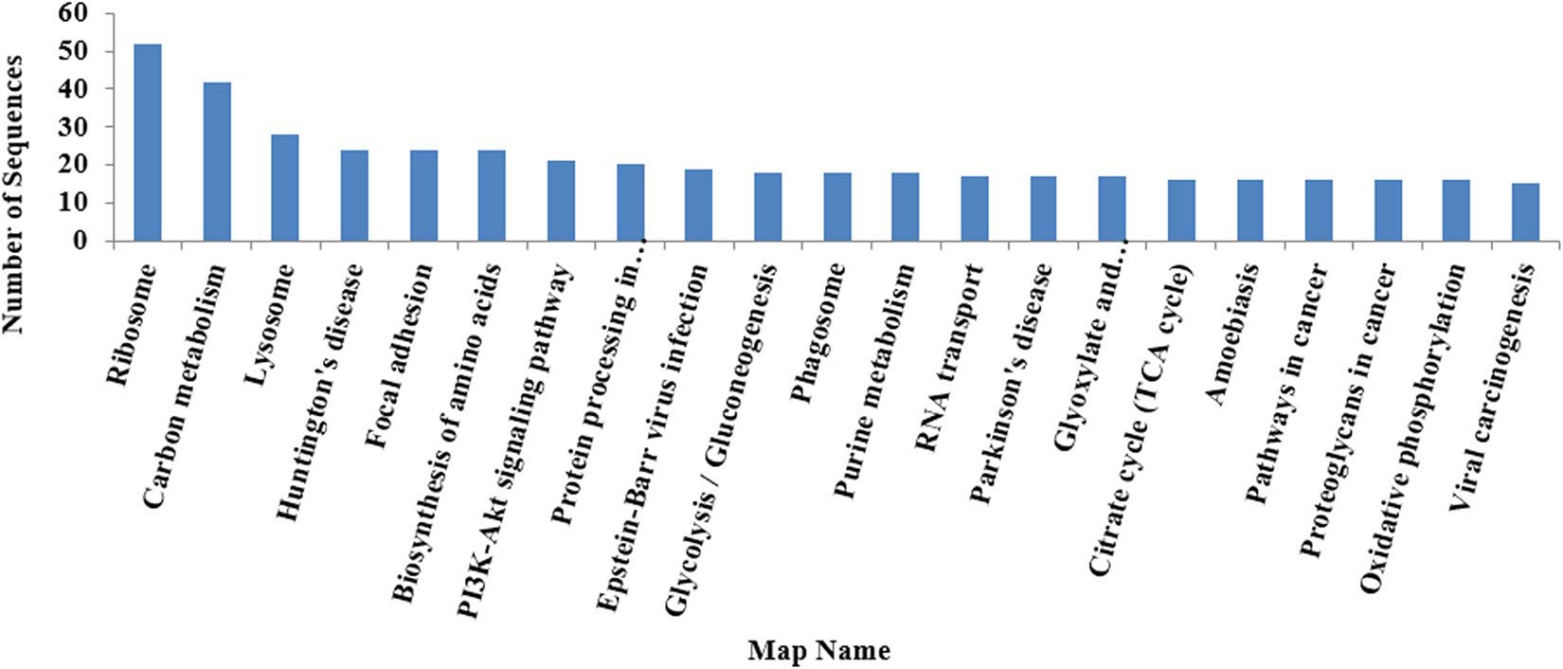
Statistics of most enriched KEGG pathways.

The KEGG pathway is used to analyze the significance level of each protein enriched in pathways, thereby determining proteins that significantly affect metabolism and signal transduction. The differentially expressed proteins were analyzed by KEGG in three larval stages, statistical assessment of the significantly enriched KEGG pathways is shown in Fig. 6. The results showed that the most significantly enriched KEGG pathways were the pathways related to ribosome and carbon metabolism, lysosome, focal adhesion, amino acid biosynthesis, the phosphatidylinositide 3-kinase/serine-threonine kinase (PI3K)/Akt signaling pathway, Epstein-Barr virus infection, purine metabolism, phagosome, glycolysis/gluconeogenesis, glyoxylate and dicarboxylate metabolism, and the tricarboxylic citrate cycle, in that order. The relationship between KEGG pathway and the number of protein sequence was showed in Fig. 7 and Table 5. The results showed that the ribosome pathway is the pathway that is annotated the most to protein sequence, carbon metabolism and lysosome successively.

**Table 5:** Top KEGG maps.

Map ID	Map Name	Number of Sequence
ko03010	Ribosome	52
ko01200	Carbon metabolism	42
ko04142	Lysosome	28
ko05016	Huntington’s disease	24
ko04510	Focal adhesion	24
ko01230	Biosynthesis of amino acids	24
ko04151	PI3K-Akt signaling pathway	21
ko04141	protein processing in endoplasmic reticulum	20
ko05169	Epstein-Barr virus infection	19
ko00010	glycolysis/gluconeogenesis	18
ko04145	phagosome	18
ko00230	purine metabolism	18
ko03013	RNA transport	17
ko05012	Parkinson’s disease	17
ko00630	glyoxylate and dicarboxylate metabolism	17
ko00020	citrate cycle (TCA cycle)	16
ko05146	amoebiasis	16
ko05200	pathways in cancer	16
ko05205	proteoglycans in cancer	16
ko00190	oxidative phosphorylation	16
ko05203	viral carcinogenesis	15

### KEGG Pathway

The KEGG pathway database (www.kegg.jp) was accessed using the KEGG automatic annotation server. Some of the significantly enriched KEGG pathways are shown in Supplementary Figures 1–3, including the ribosome (map03010), carbon metabolism (map01200), and lysosome (map04142) pathways, respectively in website www.kegg.jp. The KEGG pathways related to early development are shown in Supplementary Figures 4–12, including the PI3K/Akt signaling pathway (map04151), ErbB signaling pathway (map04012), mTOR signaling pathway (map04150), glycolysis/gluconeogenesis (map00010), biosynthesis of amino acids (map01230), protein digestion and absorption (map04974), focal adhesion (map04510), the gamma-aminobutyrick acid-ergic (GABAergic) synapse (map04727), and phagosomes (map04145), respectively in website www.kegg.jp.

### Predicted interactions of identified DEPs

**Figure 8:**
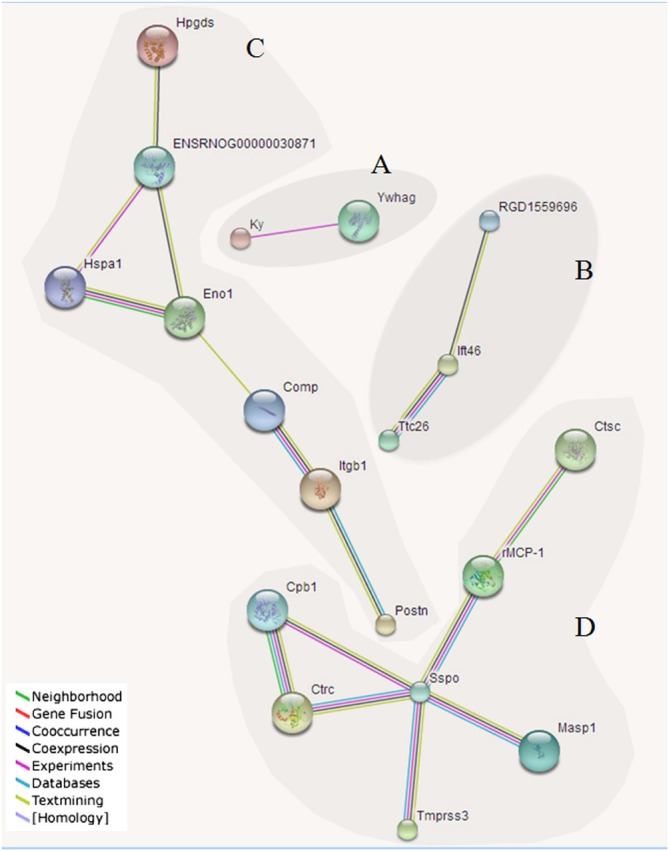
Predicted interactions of identified DEPs. Different line colours represent types of evidence for association. Four major clusters associated with neuron development (**A**), cilium assembly and motor protein (**B**), calcium and metal-binding (**C**), hydrolase (**D**). Proteins without interactions have been removed from the graph. Protein abbreviations and corresponding full name are shown: Transmembrane protease serine 3 (Tmprss3); 14-3-3 protein gamma (Ywhag); Mannan-binding lectin serine protease 1(Masp1); Hematopoietic prostaglandin D synthase (Hpgds); Kyphoscoliosis peptidase (Ky); Integrin beta-1 (Itgb1); Periostin precursor (Postn); Chymotrypsin-C (Ctrc); Intraflagellar transport protein 46 homolog (Ift46); Dipeptidyl peptidase 1 (Ctsc); Alpha-enolase (Eno1); Mast cell protease 1(rMCP-1); Tetratricopeptide repeat protein26 (Ttc26); SCO-spondin (Sspo); Calmodulin (ENSRNOG00000030871); Carboxypeptidase B precursor (Cpb1); Kinesin-like protein KLP6(RGD1559696); Cartilage oligomeric matrix protein precursor (Comp); Heat shock 70 kDa protein 1 A/1B (Hspa1). Nodes: Network nodes represent proteins; Node Size: small nodes and large nodes; Node Color: colored nodes: query proteins and first shell of interactors; white nodes: second shell of interactors; Edges: Edges represent protein-protein associations; Network Stats: average node degree: 0.642; avg. local clustering coefficient: 0.241; expected number of edges: 7; protein-protein interaction (PPI) enrichment p-value: 0.000792.

**Figure 9:**
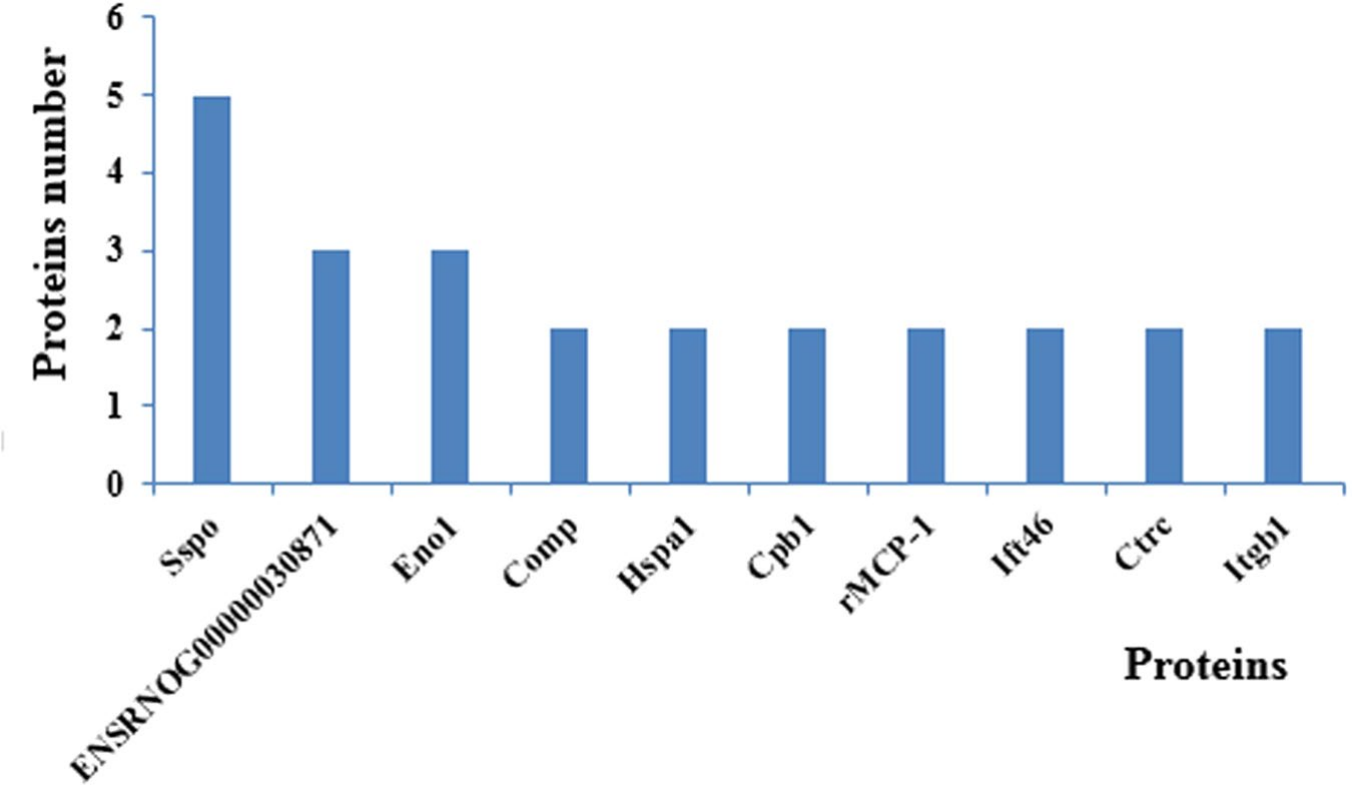
Number of interactive network of protein co-expression.

Figure 8 was obtained from the http://string.embl.de/ website, and shows the predicted interactions of the identified DEPs. Four major clusters were associated with neuron development (A), cilium assembly and motor protein (B), calcium and metal-binding (C), hydrolase (D).The number of interactive networks of co-expressed proteins is shown in Fig. 9. The identified differentially expressed co-expressed proteins mainly included transmembrane protease serine 3 (Tmprss3), 14-3-3 protein gamma (Ywhag), mannan-binding lectin serine protease 1 (Masp1), hematopoietic prostaglandin D synthase (Hpgds), kyphoscoliosis peptidase (Ky), integrin beta-1 (Itgb1), periostin precursor (Postn), chymotrypsin-C (Ctrc), intraflagellar transport protein 46 homolog (Ift46), dipeptidyl peptidase 1(Ctsc), alpha-enolase (Eno1), mast cell protease 1 (rMCP-1), tetratricopeptide repeat protein26 (Ttc26), SCO-spondin, (Sspo); calmodulin, (ENSRNOG00000030871); carboxypeptidase B precursor, (Cpb1); kinesin-like protein 6,(RGD1559696); cartilage oligomeric matrix protein precursor (Comp), and heat shock 70 kDa protein 1 A/1B (Hspa1) (Fig. 8).

### mRNA expression of the 17 differentially expressed genes

**Figure 10:**
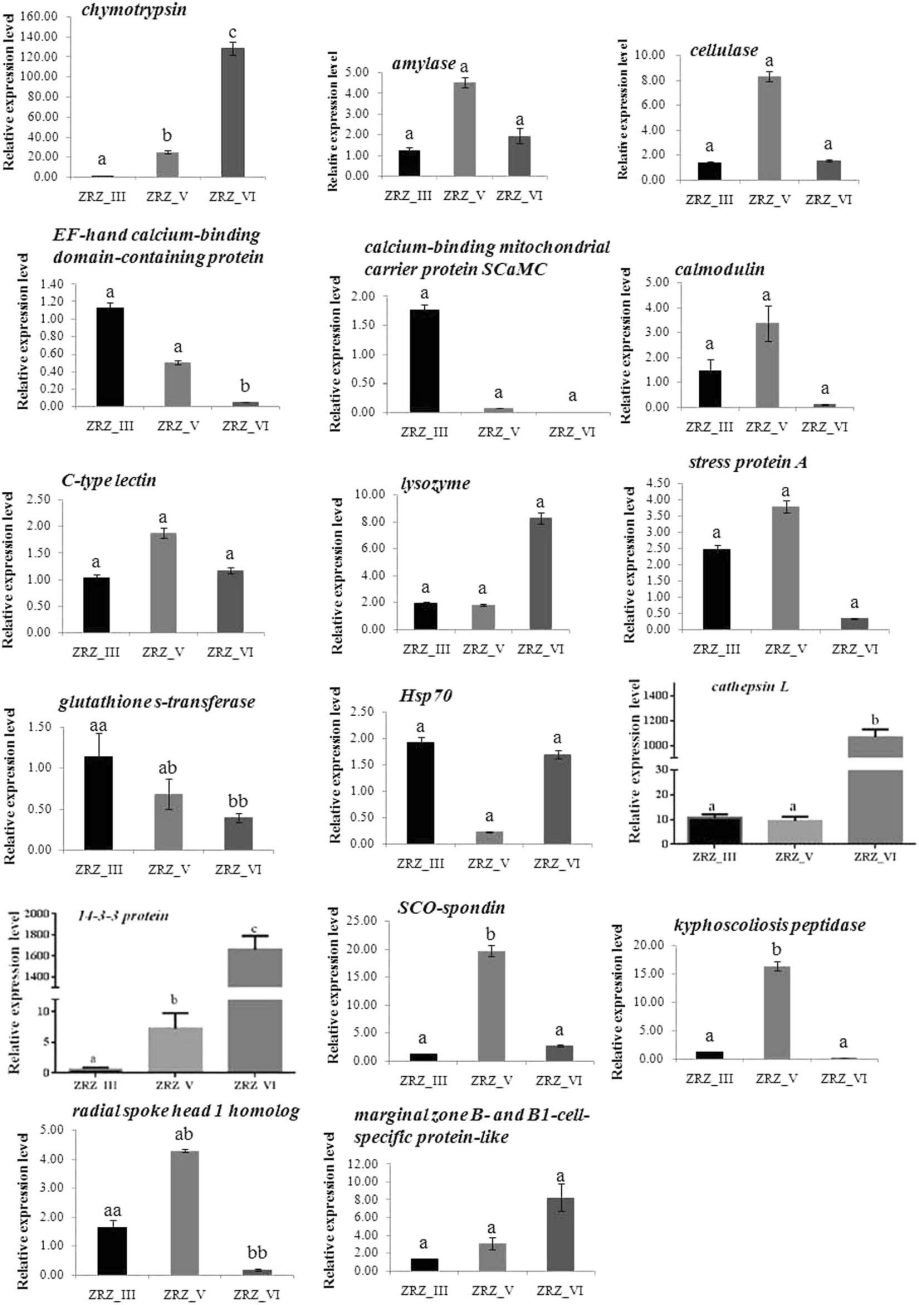
The mRNA expression of 17 DEPs. Different letters (for example (**a**–**c**)) indicate that variations were statistically different by Student’s *t*-test analysis (p < 0.05). In addition, if one of the two letters (aa, ab, bb) is the same, there is not statistically different.

To investigate the transcriptional levels of the identified proteins, 17 of the expressed sequence tags matched by proteins, namely chymotrypsin, amylase, cellulase, EF-hand calcium-binding domain-containing protein, calcium-binding mitochondrial carrier protein SCaMC, calmodulin, C-type lectin, lysozyme, stress protein A, glutathione s-transferase (GST), Hsp70, cathepsin L, 14-3-3 protein, SCO-spondin, kyphoscoliosis peptidase, radial spoke head 1 homolog, marginal zone B- and B1-cell-specific protein-like, were chosen for real-time PCR (qPCR) analysis. The melting curve analysis of the PCR products revealed only one melting temperature peak for each amplification reaction, ensuring the specificity of each primer pair. The qPCR results showed that chymotrypsin, cathepsin L, and 14-3-3 protein were upregulated from ZRZ_III to ZRZ_VI, whereas two genes EF-hand calcium-binding domain-containing protein and GST were downregulated at this stage. SCO-spondin, kyphoscoliosis peptidase, and radial spoke head 1 homolog were upregulated at the ZRZ_VI stage, and the expression levels of the remaining gene were not statistically different among the three stages (p > 0.05) (Fig. 10). Compared with the protein expression patterns, the results are shown in Table 6. The mRNA expression exhibited similar patterns with proteins, whereas EF-hand calcium-binding domain-containing protein, cathepsin L, 14-3-3 protein, and SCO-spondin had different patterns (Table 6).

**Table 6:** Comparison of mRNA and protein expression levels for 17 genes.

NO	Description	Expression pattern of mRNA	Expression pattern of proteins
1	chymotrypsin	ZRZ_VI > ZRZ_V > ZRZ_III	ZRZ_VI > ZRZ_V > ZRZ_III
2	amylase	ZRZ_V > ZRZ_VI > ZRZ_III	ZRZ_V > ZRZ_VI > ZRZ_III
3	cellulase	ZRZ_V > ZRZ_VI > ZRZ_III	ZRZ_V > ZRZ_VI > ZRZ_III
4	EF-hand calcium-binding domain-containing protein	*ZRZ_III > ZRZ_V > ZRZ_VI	ZRZ_V > ZRZ_VI = ZRZ_III
5	calcium-binding mitochondrial carrier protein SCaMC	^a^ZRZ_III > ZRZ_V > ZRZ_VI	ZRZ_III > ZRZ_VI > ZRZ_V
6	calmodulin	^b^ZRZ_V > ZRZ_III > ZRZ_VI	ZRZ_III > ZRZ_V = ZRZ_VI
7	C-type lectin	^a^ZRZ_V > ZRZ_VI > ZRZ_III	ZRZ_III > ZRZ_V > ZRZ_VI
8	lysozyme	^a^ZRZ_VI > ZRZ_III > ZRZ_V	ZRZ_V > ZRZ_VI > ZRZ_III
9	stress protein A	^a^ZRZ_V > ZRZ_III > ZRZ_VI	ZRZ_VI > ZRZ_V > ZRZ_III
10	glutathione s-transferase	ZRZ_III > ZRZ_V > ZRZ_VI	ZRZ_III > ZRZ_V > ZRZ_VI
11	Hsp70	^a^ZRZ_III > ZRZ_VI > ZRZ_V	ZRZ_VI > ZRZ_III = ZRZ_V
12	cathepsin L	*ZRZ_VI > ZRZ_V > ZRZ_III	ZRZ_III > ZRZ_V > ZRZ_VI
13	14-3-3 protein	*ZRZ_VI > ZRZ_V > ZRZ_III	ZRZ_V > ZRZ_VI = ZRZ_III
14	SCO-spondin	*ZRZ_V > ZRZ_VI > ZRZ_III	ZRZ_VI > ZRZ_V = ZRZ_III
15	kyphoscoliosis peptidase	ZRZ_V > ZRZ_III > ZRZ_VI	ZRZ_V > ZRZ_III > ZRZ_VI
16	radial spoke head 1 homolog	^b^ZRZ_V > ZRZ_III > ZRZ_VI	ZRZ_III > ZRZ_V > ZRZ_VI
17	marginal zone B- and B1-cell-specific protein-like	ZRZ_VI > ZRZ_V > ZRZ_III	ZRZ_VI > ZRZ_V > ZRZ_III

Note: The letter “a” showed *P* > 0.05 between three development stages; the letter “b” showed *P* > 0.05 between ZRZ_III and ZRZ_V; the symbol “*” showed different variations between proteins and mRNA expression pattern.

## Discussion

This study mainly focused on the three developmental stages of *B*. *areolata*, namely the veliger stage of larval *B*. *areolata* before and after attachment, and the juvenile stage. When entering the veliger prior to settlement stage (described as metamorphosing larvae), with the loss of velum and gill development, the larvae crawled with the developed foot and began their feeding. When morphological changes occurred, including the transition stage from planktonic larvae to benthic life and from the later veliger stage to the juvenile stage, the feeding habits of *B*. *areolata* also gradually changed from phytophagy to sarcophagy.

Gastropods metamorphosis is important to understand the life and evolution of the species. In this study, we identified over a thousand proteins differentially expressed before and after metamorphosis. The results revealed two thirds genes of 17 differentially expressed proteins exhibited the same trends at the mRNA level certification experiment, the potential function of some differentially expressed proteins in transition of the gastropods lifestyle were discussed.

### Proteins Related to the Metabolism in GO Functional Annotations

In the BP-classified enrichment, the proportion of proteins related to metabolism was the largest (Fig. 4). According to GO functional annotations, the number of proteins involved in the metabolism of these molecules including dynein, ATPase, spectrin, and hemocyanin was the largest. These proteins activate important metabolic and cellular processes of larval *B*. *areolata* at the early development stage, thereby allowing the efficient and orderly development and metamorphosis of the larvae. Dynein is a high molecular weight protein that is distributed in nervous tissues and organs that function in the transport of metabolites^34^ and may transfer vesicles along the microtubules, showing its important role in intracellular vesicular transport^35^. Dynein itself may not enable the transfer of vesicles or organelles, but may function with ATPase and dynactin^36^. Furthermore, dynein may regulate the proliferation and cycle of nerve cells, thereby promoting development of the nervous system^37^. There have been studies on distribution in the optic nerve system of molluscs^38^, the left and right spiral formations^39^, and reproductive development^28^. Spectrin, a cytoskeletal protein, may maintain the stability of membrane and its shape and participate in a variety of MFs^40^, and may also interact with actin to establish a membrane network that maintains the elasticity of the cell^41^. In addition, because spectrin is a multifunctional protein that plays a multifaceted role, it may participate in signal transduction, cell cycle, intracellular transport, immunological reaction, and other MFs. *H*. *rufescens*^42^, *A*. *californica*^43^ and other molluscs have also been investigated. Hemocyanin, a respiratory protein, mainly transports oxygen to some invertebrates^44^. This protein is a complex structure in the hemolymph of gastropod and is essential for the circulatory and respiratory systems of gastropods. Hemocyanin also plays an important role in respiration and the immunological reactions of gastropod. Moreover, in some molluscs and under certain conditions, hemocyanin may exhibit phenoloxidase activity^45,46^. Thus, this protein participates in phagocytosis, agglutination, and other immunological reactions^47^. *H*. *diversicolor*^48^ and *Rapana thomasiana*^49^ have also been studied.

### KEGG Pathway Enrichment Analysis of Differentially Expressed Proteins in Three Stages

In the KEGG pathway enrichment analysis, significantly enriched KEGG pathways were related to ribosome, carbon metabolism, and lysosomal pathways (Fig. 6), indicating that at the developmental stage of larval *B*. *areolata*, the genes and proteins participating in these related pathways were considerably expressed and regulated.

#### Ribosome pathways

The ribosome is composed of rRNA and protein^50^, which are very important in the cell. Ribosomes are at the center of protein synthesis, and their levels of cells with quick proliferation and strong secretion is higher than that in the normal cell^51^. In eukaryotic cells, ribosome synthesis is a complex dynamic process in which hundreds of diverse genes are involved^52^. The results of this study showed that during the early development and metamorphosis of larval *B*. *areolata*, the genes and proteins that participate in the ribosome pathway are active and the ribosome is closely related to intracellular protein synthesis. This observation proved that in the early developmental stage, larval *B*. *areolata* may regulate various physiological and biochemical processes related to protein synthesis by regulating the genes participating in the ribosome pathway. Studies on *Papana venosa*^53^, *H*. *diversicolor*^54^, and some other gastropods have also been reported.

#### Carbon metabolism pathways

Carbon metabolism is the most basic of life activities, as it is an important pathway for energy metabolism in organisms. This pathway involves glycolysis, phosphopentose pathway, citric acid cycle, six carbon fixation processes, and methanol metabolism^55^. Carbohydrates provide energy and raw materials for embryonic development^56^. In this study, the signaling pathway related to glycolysis or gluconeogenesis was significantly enriched. In addition, many tissues and organs are formed and constructed during development; the fact that enrichment of the signaling pathway, in which DEPs participated, is related to the synthesis of amino acid also illustrated this point. The signaling pathway related to the digestion and absorption of protein was also significantly enriched, probably due to transition of the larval *B*. *areolata* from phytophagy to sarcophagy.

#### Lysosomal pathways

Lysosomes are membrane-coated cystic cells that contain a broad range of hydrolases, which can break down a variety of macromolecules and play significant roles in apoptosis and the cellular defensive response^57^. During the developmental stage of larval *B*. *areolata*, a large number of genes and proteins participating in the carbon metabolic and lysosomal pathways are significantly differentially expressed. These results suggest that energy metabolism and cellular defense are adaptive to complex physiological changes during the developmental stage of larval *B*. *areolata*. *Patella vulgate*^58^, *Achatina achatina*^59^ and some other gastropods were also investigated.

#### Pathways related to cytoskeleton, cell adhesion and ECM

“Skeletons” exist inside and outside of cells, including intracellular, transmembrane and extracellular components, “Skeletons” mainly refer to cytoskeleton, cell adhesion and ECM, respectively, and comprise an important network. KEGG analysis showed that focal adhesion was significantly enriched. Focal adhesion is a type of adhesive contact between the cell and extracellular matrix (ECM), which are mediated by integrins, resulting in dynamic cell anchoring connections^60^. The assembly and depolymerization of focal adhesions affect the cell shape and site and direction of pseudopods. In addition, extracellular and intracellular signal synergies regulate the formation, distribution, and turnover of focal adhesion spatially and temporally, which consequently affect cell migration. During cell migration, focal adhesion is a constant dynamic structure in the continuous links of assembly, depolymerization, and reassembly. Integrin, acting as a bridge, is a small and unstable adhesive complex that links ECM and intracellular protein formation^61^. When a large number of proteins are attracted to the adhesive complex, large and stable focal adhesions are formed. Cell mitosis is related to cell proliferation and differentiation, embryonic development, and tissue and organ formation. A series of processes such as integrin-mediated extracellular adhesion, focal adhesion assembly, stress fiber arrangement, and mitotic spindle orientation affect the mitosis of adherent mammalian cells. Myosin and dynein play important roles in these processes. In this study, the signaling pathway related to focal adhesion was significantly enriched and closely related to cell division in embryonic development. Moreover, myosin, dynein, and other proteins were identified. ECM receptors interact with the relevant pathway that is significantly enriched. The differentially expressed proteins are involved in functions of cytoskeleton, cell adhesion, and ECM in three development stages. These proteins may contribute to the tissue remodeling during metamorphosis, for example, degradation of velum, foot and transitions of muscular and nervous systems.

ECMs are composed of a complex mixture of proteoglycan, glycoprotein, aminoglycan, and other biomolecules; can interact with the aid of their surface receptors; and play important roles in cell adhesion, migration, differentiation, and proliferation^62^. The ECMs have potential roles in tissue remodeling, ECM remodeling has been proved essential in metamorphosis of amphibian^63,64^, insects^65^, and bivalve *C*. *gigas*^13^. This study result speculate that ECMs may function in regulating cell fates in metamorphosis of *B*. *areolata*. In this study, the pathway that related to the interaction of ECM receptors may complement each other with that related with focal adhesion and bridge cell adhesions well. The PI3K/Akt signaling pathway, which regulates cell metabolism and participates in the survival and antiapoptosis of cells, was also enriched. This pathway also plays an important role in the early embryonic development of mammalians^66^, and regulates embryonic stem cells^67^. Therefore, enrichment of this signaling pathway may considerably affect the development process of *B*. *areolata*.

### Cluster Analysis of the Three Larval Stages of Development and Metamorphosis

Cluster analysis results showed that in the horizontal direction, the protein expression modes at ZRZ_III and ZRZ_V were of the same branch, and that the expression mode at ZRZ_VI was of another branch at the three stages of early development of larval *B*. *areolata*. This result is consistent with the developmental characteristics of larval *B*. *areolata* and is more similar to those at ZRZ_III and ZRZ_V of the veliger stage than that at ZRZ_VI. Irreversible metamorphosis is necessary from the veliger stage to the juvenile stage; each larval undergoes a certain degree of change, which is largely reflected in the protein expression mode. Therefore, the cluster analysis results were consistent with the larval developmental process. In the vertical direction, DEPs were divided into two major branches. The protein expression in the first branch was upregulated with developmental stage. In addition, the proteins mainly participated in the formation of actin filament and corresponding biological process including cell adhesion, cytoskeleton construction, and ion transport. Actin is closely related with the growth of molluscs, and its genes are common in transcriptomics studies on the early development of molluscs. It mainly participates in actin filament formation, cell proliferation, intracellular signal transduction, as well as some other processes^68^. Proteins that participate in cell adhesion play important roles in important physiological processes, including cell migration, growth, stress tolerance, and immunity^69^. Thus, the enrichment of each protein involved in physiological activities such as the cell migration and growth exhibited a down-and-up trend. The result showed that at the veliger stage, the physiological activities such as the cell migration and growth of *B*. *areolata* larvae were slightly weaker than those from the metamorphosis stage to the juvenile stage. The protein expression in the second branch was downregulated, and the proteins mainly participated in gene expression, protein synthesis and modification, and ribosome biosynthesis. In the development process from the early veliger to late stage, various parts of the organs of *B*. *areolata* larvae grow quickly. Consequently, the expression of proteins related to cell growth was upregulated. Ribosomal proteins were highly enriched in the *B*. *areolata* larvae in the early stage of development, as they participate in the synthesis and modification of proteins^70^ that satisfy the needs of the rapid growth of larvae. This result is similar to findings on *A*. *californica*^71^. After larval metamorphosis is complete, the types of proteins in various body parts of this organism slightly change. Therefore, the expression of proteins related to protein synthesis and modification may be downregulated. Similar results have been observed in *Lottia gigantean*^72^ and *H*. *rufescens*^42^.

### Proteins Associated with Shell Formation, Body Torsion, Changes in the Feeding Habits, Attachment and Metamorphosis, and Immune Responses in *B*. *areolata* Larvae

#### Proteins related to changes in feeding habits

In this study, a wide range of lipases, amylases, and proteases were identified. The digestive enzymes were diverse, consistent with the fact that *B*. *areolata* at different stages use different foods as a source of nutrition. The expression levels of these digestive enzymes varied at different developmental stages, showing the importance of regulating protein levels during adaption to changes in the feeding habits. In terms of the serine proteases, represented by trypsin and chymotrypsin, studies on crustaceans have shown that trypsin and chymotrypsin are the two most active proteases in the digestive gland, which account for more than 60% of the protein digestion in prawns^73^. In this study, the levels of chymotrypsin were in the the order of ZRZ_VI > ZRZ_V > ZRZ_III, and same trend was observed at the mRNA level (Fig. 10). This is consistent with previous study that a chymotrypsin gene was highly induced during the metamorphosis of *H*. *rufescens* in the digestive system^74^ and larva metamorphosis of *C*. *gigas*^13^, these results may show a transition of the digestive system in metamorphosis. Additionally, there were different levels of carboxypeptidase and serine proteinase, and levels of amylase and cellulase were in the order of ZRZ_ V > ZRZ_ VI > ZRZ_III (Supplementary Tables 1–3); the genes exhibited the same trend (Fig. 10). There were also different levels of esterase and chitinase, which are thought to be closely related to changes in the feeding habits of the larval *B*. *areolata*. Agrawal *et al*.^75^ compared the activities of proteases, amylases, and lipases in carnivorous fish Wallago, omnivorous fish Clarias, and herbivorous fish Labeo, and found that the activity of carbohydrases is higher in the Labeo (herbivorous fish) than in the Wallago (carnivorous fish) and Clarias (omnivorous fish). Regarding proteases, maximum activity was found in Wallago (carnivorous fish). The difference in lipase activity was not as pronounced. Proteases and lipases exhibited the same activity in the order of carnivorous > omnivorous > herbivorous. Conversely, amylase activity was in the order of herbivorous > omnivorous > carnivorous. These results showed that there is a correlation between the normal diet of fish and the relative activity of digestive enzymes^75^. These findings are similar to the results of this study, which indicated that the activities of different digestive enzymes correlated with different feeding habits.

#### Immune-related protein at the metamorphosis stage

Mollusks lack an adaptive immune system, and mainly depend on the innate immune response as a defense against various pathogens. In this study, a variety of immune proteins were identified including C-type lectin, lysozyme, stress protein A, GST, Hsp70, cathepsin L and marginal zone and B1 B cell-specific protein-like (as part of the natural immune memory). C-type lectin, GST, and cathepsin L levels were in the order of ZRZ_III > ZRZ_V > ZRZ_VI, and lysozyme was highly expressed at ZRZ_V. The abundance of stress protein A was in the order of ZRZ_VI > ZRZ_V > ZRZ_III. Hsp70 was only detected at ZRZ_VI (Supplementary Tables 1–3). Regarding the mRNA expression level, cathepsin L was upregulated from ZRZ_III to ZRZ_VI, the inconsistency at the mRNA and protein levels provokes that investigations are needed to reveal the changes of cathepsin L genes during metamorphosis. GST was downregulated from ZRZ_III to ZRZ_VI. The expression levels of C-type lectin, lysozyme, stress protein A, Hsp70, marginal zone and B1 B cell-specific protein-like genes were not statistically different among the three stages (p > 0.05), C-type lectin and stress protein A were slightly upregulated at the ZRZ_V stage. Metamorphosis is the critical stage when larvae increase the expression of immune-related genes and respond to environmental stimulus^1^. Balseiro *et al*.^1^ showed that at this metamorphic stage in *Mytilus galloprovincialis*, the expression levels of immune-related genes were higher than those in oocytes, suggesting that immune-related genes are active in mussel larvae. The upregulation of innate immune-related genes during metamorphosis was previously reported in ascidian *Boltenia villosa*^76^. Protein expression began to increase after the veliger stage to prepare larvae for settlement. The activation of innate immunity genes during metamorphosis may not only reflect maturation of the innate immune system but also might correlate with the resorption and re-structuring of larval tissues, as well as the ability of larval to detect and respond to bacterial settlement cues^1,76^. C-type lectins play important roles in the immunity of invertebrate. Intracellular lectins mainly play roles in protein trafficking and sorting, whereas extracellular lectins are important for cell signaling and pathogen recognition^77^. Bao *et al*.^78^ investigated the expression of putative C-type lectin fold (MyCLF) during embryonic development from the Japanese scallop *Mizuhopecten yessoensis*. The authors found that the expression level increased in the gastrulae, trochophore, and early D-shaped larvae (veliger stage), and then decreased in D-shaped larvae, and increased hundreds of times in metamorphosing larvae. In another study, lysozymes were selected for expression profile analysis throughout the different developmental stages (trochophore, veliger, metamorphosis, post-settlement, and spat) in mussel *M*. *galloprovincialis*, and it was observed that expression of lysozyme genes increased during the transition from trochophore to spat^1^. In this study, C-type lectin and lysozyme were highly expressed at ZRZ_III and ZRZ_VI, respectively, similar to previous studies. In addition, Hsps have dual functions, and are involved in the stress response and developmental processes^79^. Gunter *et al*.^79^ observed higher levels of Hsp70 expression in tissues undergoing larval morphogenesis in the marine gastropod *H*. *asinina*. Hsp70 is expressed in unique and overlapping patterns in the prototroch, foot, and mantle, and returns to lower levels after morphogenesis is complete. These patterns of Hsp70 expression in *H*. *asinina* are similar to those observed in ecdysozoans and deuterostomes^79^. In this study, Hsp70 was only detected at ZRZ VI, akin to previous research studies.

#### Proteins related to attachment and metamorphosis

In marine invertebrates, there are usually several metamorphosis events in early embryo development, which include development of the larvae internal structure, external morphology, physiological processes, and behavior. In planktonic life at the early veliger stage to the benthic life with gradually disappearing velum, the larvae undergo the attachment process. The nervous system plays an important role in the activation and regulation of the larval metamorphosis. GABA is a neurotransmitter that has been widely used in the chemical induction of marine shellfish larvae metamorphosis^80^. Fang *et al*.^81^ proposed in their studies on larval *Arca granosa* L. that tryptophan, GABA, and acetyl choline within certain concentration ranges exert clear inducing effects. GABA may significantly induce the larval attachment of *H*. *discus*^82^, which is consistent with the findings of Morse *et al*.^83^ on *H*. *rufescens*. GABA induced both settlement and metamorphosis in the mussel *M*. *galloprovincialis*, the clams *Venerupis pullastra* and *Ruditapes philippinarum*, and the oyster *Ostrea edulis*^84^. In this study, the GABA-ergic synaptic signaling pathway was also significantly enriched (Fig. 6), which was likely related to larval attachment. The immune-related genes may be involved in the interaction with biofilm during settlement (i.e., attachment to a surface and metamorphosis into juveniles) when the larvae are searching for the best habitat to settle. Some studies have discussed the processes of metamorphosis in some marine organisms, and lectins (sugar binding proteins and glycoproteins) have been suggested to be involved in settlement, metamorphosis, and tissue remodeling^76,85–87^. Maki and Mitchell^88^ showed that lectins are involved in the settlement and metamorphosis of marine invertebrate larvae, and proposed a biochemical lectin model for settlement and metamorphosis of some marine invertebrate larvae. The authors demonstrated that lectin on the surface of larvae “recognizes” and binds to a glycoconjugate in the exopolymer of marine bacteria. Matsutani *et al*.^89^ showed that lectin-like factors are involved in larval settlement and metamorphosis in the abalone *H*. *discus hannai*. Bao *et al*.^78^ suggested that MyCLF (a putative C-type lectin fold) may play important roles during the metamorphosis phase of *M*. *yessoensis*.

#### Proteins related to shell formation

Changes in shell formation and body shape are important events in the development of molluscs. Among EF-hand calcium-binding domain-containing protein, calcium-binding mitochondrial carrier protein SCaMC, carbonic anhydrase, and calmodulin (Supplementary Tables 1–3), it has been reported that calmodulin participates in shell formation^90^. Furthermore, a large amount of proteins related to calcium metabolism may be involved in calcium deposition. Regarding mRNA expression level, EF-hand calcium-binding domain-containing protein gene were upregulated at the ZRZ_III stage, and calcium-binding mitochondrial carrier protein SCaMC and calmodulin were slightly upregulated at the ZRZ_III and ZRZ-V stage respectively, suggesting that these genes were involved in shell formation. Whereas, in real-time PCR assay, the gene of EF-hand calcium-binding domain-containing protein had different patterns. Thus, it still remains unclear whether transcription/translation inhibitors would affect the metamorphosis of *B*. *areolata*.

#### Proteins related to body torsion and neural development

In this study, 14-3-3 and SCO-spondin were identified. Comparison of the ZRZ-III and ZRZ-V stages showed that 14-3-3 was significantly upregulated at ZRZ-V (Supplementary Tables 1–3). 14-3-3 participates in growth and development, tumorigenesis and receptor signal regulation, molecular chaperone, and activation or inhibition of the target protein activity^91–93^. The overexpression of 14-3-3 in the body plays an important role in nerve cell differentiation^94^. Studies on brain development in humans and mice showed that 14-3-3 deficiency may cause brain death or severe brain deformity^95^. In the embryonic development of frogs, 14-3-3 in the early development stage participates in determining the left and right in the axial direction of the embryo^96^. The body torsion of the larval *B*. *areolata* is likely related with 14-3-3, and 14-3-3ε is related with the neural development of *B*. *areolata*. Additionally, SCO-spondin is required for neurogenesis during early brain development, Vera *et al*.^97^ suggested that SCO-spondin is a vital embryonic cerebrospinal diffusible factor regulating the balance between proliferation and differentiation of the brain neuroepithelial cells. For 14-3-3 protein and SCO-spondin, the inconsistency at the mRNA and protein levels provokes the question of whether two gene participates in body torsion and neural development of *B*. *areolata*, that still suggest a further investigation.

#### Other proteins

In this study, the radial spoke head protein was identified, which is structural components of the axoneme and are suggested to be involved in producing flagellar patterns through regulation of the dynein arms^28^. The radial spoke head protein decreased from ZRZ_III to ZRZ_VI, indicating that it might be related to velum atrophy. Kyphoscoliosis peptidase plays a vital role in muscle growth^98^, and at the protein and mRNA level, the pattern was ZRZ_V > ZRZ_III > ZRZ_VI, indicating that it might correlate with muscle growth. We also noticed a discrepancy between the protein level and mRNA expression of the EF-hand calcium-binding domain-containing protein, cathepsin L, 14-3-3 protein, and SCO-spondin (Table 6). Thus, it still remains unclear whether transcription/translation inhibitors would affect the metamorphosis of *B*. *areolata*. Post-translational modifications may be responsible for this discrepancy. Changes at the mRNA level may not indicate changes at the protein level. Validating protein expression experiments are required to estimate the roles of transcription/translation-related genes in metamorphosis.

## Conclusions

This study provides the first comparative analysis of proteomic profiles at different embryonic development stages of *B*. *areolata*. A total of 5583 proteins were identified. The DEPs were annotated and enriched in the GO and KEGG databases. Identification of these proteins will provide a basis for exploring larval development and metamorphosis mechanisms. We identified the protein profiles of *B*. *areolata* development related to shell formation, body torsion, changes in feeding habits, attachment and metamorphosis, and immune response.

## Materials and Methods

### Sampling

*B*. *areolata* were purchased from the Dongshan farm, Fujian province, selected individuals with vitality. Snails were maintained in an ecological flow-through water pond (20.0–30.0 °C, salinity 28.3–35.1, pH 7.8–8.5, oxygenated). The snails were fed daily with oysters and chopped fresh fish. Embryos were cultured, and fertilization, hatching, and developmental conditions were measured. Three groups of larval *B*. *areolata* at different developmental stages including the middle veliger stage before attachment (ZRZ-III), later veliger stage (velum atrophy) (ZRZ-V), and juvenile stage (ZRZ-VI) were used. Each group was replicated three times. Early veliger larvas were fed daily with algae. The late veliger stage sinks to the substratum and leads a benthic life, the feeding habits also gradually changed from phytophagy to sarcophagy, the snails were fed daily with oysters. Larval *B*. *areolata* were sampled from a culture pond containing 2,000,000–3,000,000 larvae. The sampled larvae were flushed with clean seawater, transferred to a 1.5 mL centrifuge tube with a suction pipe, rinsed twice with normal saline, frozen in liquid nitrogen, and stored at −80 °C.

### Extraction and Quantification of Proteins

Samples were thawed at 4 °C. Subsequently, 60 mg sample was weighed. Then 200 μL SDT lysis buffer (4% SDS, 100 mM Tris-HCl, 1 mM DTT, pH7.6) was added to the sample, followed by solubilization for 20 s with a tissue homogenizer; this was repeated three times. Then, 500 μL SDT lysis buffer is added. The mixture was shaken and heated for 10 min in a water bath. After 30 min ultrasonic treatment, the mixture was centrifuged for 30 min at 12,000 × g and 4 °C, and the supernatant was saved for subsequent experiments. The BCA assay was used for protein quantification.

### SDS-PAGE for Protein Separation

Approximately 20 μg of each protein sample was resolved and mixed with 5× loading buffer added at 1:5 ratio. The mixture was boiled for 5 min in a water bath and centrifuged for 10 min at 14,000 × g. The supernatant was used, and the mixture was electrophoresed for 60 min on 12.5% SDS-PAGE gels at 15 mA. Protein bands were visualized by Coomassie Blue R-250 staining.

### Filter-Aided Sample Preparation

About 300 μg samples were collected and mixed with DTT until a final concentration of 100 mM was reached. The mixture was boiled for 5 min in a water bath and cooled to room temperature. Approximately 200 μL UA buffer (8 M Urea, 150 mM Tris-HCl, pH 8.0) was added and mixed well. The mixture was transferred to a 30 kDa centrifuge tube for ultrafiltration and centrifuged for 15 min at 14,000 × g, after which the filtrate was discarded. After adding 200 μL UA buffer, the mixture was centrifuged for 15 min at 14,000 × g, and the filtrate was discarded. Then IAA (50 mM IAA in UA) was added in UA buffer (100 μL), and the mixture was oscillated for 1 min at 600 rpm. The mixture was allowed to settle for 30 min in the dark at room temperature and centrifuged for 10 min at 14,000 × g. UA buffer (100 μL) was added, and the mixture was centrifuged for 10 min at 14,000 × g; the procedures were repeated twice. Next, 100 μL of NH_4_HCO_3_ buffer was added, and the mixture was centrifuged for 10 min at 14,000 × g, the procedures were repeated twice. Subsequently, 40 μL trypsin buffer (2 μg Trypsin in 40 μL NH_4_HCO_3_ buffer) was added. The mixture was oscillated for 1 min at 600 rpm and allowed to settle for 16–18 h at 37 °C. The mixture was transferred into a new collecting tube and centrifuged for 10 min at 14,000 × g, after which the filtrate was collected. The filtrate was desalinized with C18 SD Extraction Disk Cartridge and quantified at OD280.

### LC-MS/MS Analysis on the Protein Digestion Product

Approximately 2 μL product of protein digestion was used for LC-MS/MS analysis and separated using a nanoliter HPLC EASY-nLC1000 system. The mobile phase A was 0.1% formic acid solution with 2% acetonitrile, and the mobile phase B was 0.1% formic acid solution with 84% acetonitrile. The chromatographic column EASY column SC200 150 μm * 100 mm (RP-C18) was balanced with 100% A solution. The sample was loaded onto the EASY column SC001 traps 150 μm * 20 mm (RP-C18) through an automatic sampler and separated by a chromatographic column at the flow rate of 400 nL/min. The gradient elution procedure was as follows: the percentage change of the mobile phase B for 0–100, 100–108, and 108–120 min is 0–45%, 45–100%, and 100% of the linear change, respectively. After the capillary separation by HPLC, the product of protein digestion is determined using Q-Exactive mass spectrometer with the following parameters: duration: 120 min; detection method: positive ion detection; parent ion scanning scope: 300–1800 m/z; MS1 resolution at M/Z 200: 70,000; and MS2 resolution at M/Z 200: 17,500. The mass-to-charge ratios of polypeptides and polypeptide fragments are obtained by collecting 20 fragment patterns (MS2 scan, HCD) after each full scan.

### Maxquant Label-Free Quantification Analysis

The nine resulting raw LC-MS/MS files were imported to the Maxquant software (1.3.0.5) for database inquiry and LFQ label-free quantification analysis. The database was Uniprot_Bivalvia_44359_20160420.fasta (containing 44359 sequences, downloaded on April 20, 2016). The database search parameters were as follows: main search ppm:6; missed cleavage: 2; MS/MS tolerance ppm: 20; De-Isotopic: ture; enzyme: trypsin; database:

Uniprot_Bivalvia_44359_20160420.fasta; fixed modification: carbamidomethyl (C); variable modification: oxidation (M), acetyl (protein N-term); decoy database pattern: reserve; LFQ: ture; LFQ min ratio count: 1; match between runs: 2 min; peptide FDR: 0.01; protein FDR: 0.01.

### Perseus Statistics and Bioinformatics Analysis

The resulting inquiry files from Maxquant were analyzed using Perseus (1. 3.0. 4) software. The samples from each of the three stages, namely ZRZ-III, ZRZ-V, and ZRZ-VI, were prepared in triplicate. Analysis was performed by one-way ANOVA, and P < 0.05 were considered statistically significant.

### GO Functional Annotations

The steps for GO annotation were as follows^99^. Target proteins were assembled and prepared through sequence alignment and batch extraction of target protein and BLAST of GO terms (mapping). GO annotation is a process of scoring the similarity between the target sequence and the sequence to be aligned to determine the reliability of the source of the GO term and the GO term structure in directed acyclic graphs. Only the GO term that satisfies the preset score may be annotated to the target protein sequence. For the supplementary annotation augmentation, the conservative motif in the EBI database of InterProScan is aligned with the target protein. The motif-related functional information is annotated to the target protein sequence. The annotation is further supplemented with ANNEX. The links between different GO terms were established, thereby improving the accuracy of the annotation. Consequently, the GO annotation results are obtained.

### GO Enrichment Analysis on Deps

Fisher’s exact test was used to evaluate the significance level of protein enrichment under each GO term.

### KEGG Pathway Annotations

The steps were as follows. The protein sequence is aligned with the KEGG database^100^, and homologous KEGG genes are obtained. The resulting gene was screened through Bi-Directional hit rate, and the orthologous candidate genes were obtained. The KO grouped data were obtained by KO based on the probability and Heuristic scoring, and the KO ranking chart is obtained. Finally, KEGG pathway mapping were finished.

### KEGG Pathway Enrichment Analysis on Deps

The KEGG pathway enrichment analysis was similar to the GO enrichment analysis. In this analysis, the KEGG pathway was the unit, and all of the qualitative proteins were background. The significance level of protein enrichment of each pathway was calculated by Fisher’s exact test to determine the significantly affected metabolism and the signal transduction mode.

### Experimental validation using qPCR

The differentially expressed protein genes were validated by qPCR to confirm the proteomic results, which was conducted using a QuantStudio 6 Flex Real-Time PCR System with Thermo Scientific DyNAmo ColorFlash SYBR Green qPCR Kit according to manufacturer instructions. Primer sequences were designed based on each identified gene sequence using Primer Premier 6 software (Premier Biosoft, USA) (Supplementary Table 4). PCR amplification experiments were conducted in triplicate under the following conditions: Initial denaturation, 95 °C for 7 min, then denaturation 40 cycles of 95 °C for 10 s, Annealing/extension 60 °C for 30 s. The results were normalized using actin (c264956_g1; gi|404452331|gb|AFR74960.1| actin, partial [Rana clamitans]) and EF1-F (c233532_g1; gi|524916452|ref|XP_005113003.1| PREDICTED:elongation factor 1-alpha [Aplysia californica]) for each sample and the 2^−ΔΔCT^ method. The expression levels of each gene in the three developmental stages were compared using a two-sided Student’s t-test, and differences were considered statistically significant at p < 0.05.

## Electronic supplementary material

Supplementary figures and tables

## References

[1] Balseiro P, Moreira R, Chamorro R, Figueras A, Novoa B (2013). Immune responses during the larval stages of *Mytilus galloprovincialis*: metamorphosis alters immunocompetence, body shape and behavior. Fish Shellfish Immun.

[2] USEPA. Methods for measuring the acute toxicity of effluents and receiving waters to freshwater and marine organisms, 5th Edition, EPA-821-R-02-012 (2002).

[3] Bishop CD, Huggett MJ, Heyland A, Hodin J, Brandhorst BP (2006). Interspecific variation in metamorphic competence in marine invertebrates: the significance for comparative investigations into the timing of metamorphosis. Integr Comp Biol.

[4] Gaume B (2011). Biomineralization markers during early shell formation in the European abalone *Haliotis tuberculata*, Linnaeus. Mar Biol.

[5] Gaume B (2014). Characterisation and expression of the biomineralising gene *Lustrin A* during shell formation of the European abalone *Haliotis tuberculata*. Comp Biochem Phys B.

[6] Jackson DJ, Degnan SM, Degnan BM (2012). Variation in rates of early development in *Haliotis asinina* generate competent larvae of different ages. Front Zool.

[7] Degnan SM, Degnan BM (2010). The initiation of metamorphosis as an ancient polyphenic trait and its role in metazoan life-cycle evolution. Phil Trans R Soc B.

[8] Williams EA, Degnan SM (2009). Carry-over effect of larval settlement cue on postlarval gene expression in the marine gastropod *Haliotis asinina*. Mol Ecol.

[9] Underwood A, Keough MJ (2001). Supply-side ecology: the nature and consequences of variations in recruitment of intertidal organisms. Unknown.

[10] Liu H (2007). Identification and characterization of a biomineralization related gene PFMG1highly expressed in the mantle of *Pinctada fucata*. Biochemistry.

[11] Dyachuk VA, Plotnikov SV, Odintsova NA (2005). Appearance of Muscle Proteins in Ontogenesis of the Mussel *Mytilus trossulus* (Bivalvia). Russ J Mar Biol/Biol Morya.

[12] Ellis I, Kempf SC (2011). Characterization of the central nervous system and various peripheral innervations during larval development of the oyster *Crassostrea virginica*. Invertebr Biol.

[13] Huan P, Wang H, Liu B (2015). A label-free proteomic analysis on competent larvae and juveniles of the pacific oyster *Crassostrea gigas*. Plos One.

[14] Lü W (2016). Evaluation of crosses between two geographic populations of native chinese and introduced thai spotted ivory shell, *Babylonia areolata*, in southern China. J World Aquacult Soc.

[15] Fu, J. *et al*. Comparative assessment of the genetic variation in selectively bred generations from two geographic populations of ivory shell (*Babylonia areolata*). *Aquac. Res*. **48** (2017).

[16] Di G, Zhang Z, Ke C (2013). Phagocytosis and respiratory burst activity of haemocytes from the ivory snail, *Babylonia areolata*. Fish Shellfish Immun.

[17] Huang R, Huang BW, Tang WJ, Chen ZC (2010). Morphological observation of the early developmental stages of *Babylonia areolata*. Journal of Oceanography in Taiwan Strait..

[18] Fiedler TJ (2010). The transcriptome of the early life history stages of the California Sea Hare *Aplysia californica*. Comp Biochem Physiol Part D Genomics Proteomics.

[19] Huan P, Wang H, Dong B, Liu B (2012). Identification of differentially expressed proteins involved in the early larval development of the Pacific oyster *Crassostrea gigas*. J Proteomics.

[20] Lopez JL (2005). Role of proteomics in taxonomy: the *Mytilus* complex as a model of study. J Chromatogr B.

[21] Swanson WJ, Vacquier VD (2002). The rapid evolution of reproductive proteins. Nat Rev Genet.

[22] Shu L, Suter MJF, Räsänen K (2015). Evolution of egg coats: linking molecular biology and ecology. Mol Ecol.

[23] Marie B (2011). Proteomic identification of novel proteins from the calcifying shell matrix of the manila clam *Venerupis philippinarum*. Mar Biotechnol.

[24] Marie B, Zanella-Cléon I, Guichard N, Becchi M, Marin F (2011). Novel proteins from the calcifying shell matrix of the pacific oyster *Crassostrea gigas*. Mar Biotechnol.

[25] Bédouet L (2012). Proteomic strategy for identifying mollusc shell proteins using mild chemical degradation and trypsin digestion of insoluble organic shell matrix: a pilot study on *Haliotis tuberculata*. Mar Biotechnol.

[26] Yan M (2015). Integration of transcriptomic and proteomic approaches provides a core set of genes for understanding of scallop attachment. Mar Biotechnol.

[27] Sun J, Zhang Y, Thiyagarajan V, Qian P, Qiu JW (2010). Protein expression during the embryonic development of a gastropod. Proteomics.

[28] Mendoza-Porras O (2014). Exploiting genomic data to identify proteins involved in abalone reproduction. J Proteomics.

[29] Mok FS, Thiyagarajan V, Qian P-Y (2009). Proteomic analysis during larval development and metamorphosis of the spionid polychaete *Pseudopolydora vexillosa*. Proteome Sci.

[30] Zhang H (2011). Quantitative proteomics identify molecular targets that are crucial in larval settlement and metamorphosis of *Bugula neritina*. J Proteomeb Res.

[31] Thiyagarajan V, Qian PY (2008). Proteomic analysis of larvae during development, attachment, and metamorphosis in the fouling barnacle, *Balanus amphitrite*. Proteomics.

[32] Thiyagarajan V, Wong T, Qian P-Y (2009). 2D gel-based proteome and phosphoproteome analysis during larval metamorphosis in two major marine biofouling invertebrates. J Proteome Res.

[33] Di G (2017). Proteomic analysis of trochophore and veliger larvae development in the small abalone *Haliotis diversicolor*. BMC Genomics.

[34] Williams JC, Xie H, Hendrickson WA (2005). Crystal structure of dynein light chain TcTex-1. J Biol Chem.

[35] Yeh T, Peretti D, Chuang J, Rodriguez-Boulan E, Sung C (2006). Regulatory dissociation of Tctex-1 light chain from dynein complex is essential for the apical delivery of rhodopsin. Traffic.

[36] Kardon JR, Vale RD (2009). Regulators of the cytoplasmic dynein motor. Nat Rev Mol Cell Bio.

[37] Nagano F (1998). Interaction of Doc2 with tctex-1, a light chain of cytoplasmic dynein. Implication in dynein-dependent vesicle transport. J Biol Chem.

[38] Martinez JM (2000). Distribution of tubulin, kinesin, and dynein in light- and dark-adapted octopus retinas. Visual Neurosci.

[39] Grande C, Patel NH (2009). Nodal signalling is involved in left-right asymmetry in snails. Nature.

[40] Patelhett S (2011). The spectrin-based membrane skeleton stabilizes mouse megakaryocyte membrane systems and is essential for proplatelet and platelet formation. Blood.

[41] Hartwig JH (1995). Actin-binding proteins.1: Spectrin super family. Protein Profile.

[42] Jiddu B, Peng Z, Asaro RJ, Qiang Z (2011). Macromolecular structure and viscoelastic response of the organic framework of nacre in *Haliotis rufescens*. Theor App Mech.

[43] Sun Y, Monje FJ, Pollak DD, Lubec G (2011). A first partial *Aplysia californica* proteome. Amino Acids.

[44] Herskovits TT (1988). Recent aspects of the subunit organization and dissociation of hemocyanins. Comp Biochem Phys B.

[45] Decker H, Ryan M, Jaenicke E, Terwilliger N (2001). SDS-induced phenoloxidase activity of hemocyanins from *Limulus polyphemus*, *Eurypelma californicum*, and *Cancer magister*. J Biol Chem.

[46] Siddiqui NI, Préaux G, Gielens C (2004). Intrinsic and induced o-diphenoloxidase activity of beta-hemocyanin of *Helix pomatia*. Micron.

[47] Coates CJ, Nairn J (2014). Diverse immune functions of hemocyanins. Dev Comp Immunol.

[48] Zhang Q (2013). Cryo-EM structure of a molluscan hemocyanin suggests its allosteric mechanism. Structure.

[49] Idakieva K (2009). Influence of limited proteolysis, detergent treatment and lyophilization on the phenoloxidase activity of *Rapana thomasiana* hemocyanin. Int J Biol Macromol.

[50] Dahlberg AE (2001). The ribosome in action. Science.

[51] Cech TR (2000). The ribosome is a ribozyme. Science.

[52] Fromont-Racine M, Senger B, Saveanu C, Fasiolo F (2003). Ribosome assembly in eukaryotes. Gene.

[53] Song H (2016). De novo transcriptome sequencing and analysis of *Rapana venosa* from six different developmental stages using Hi-seq. 2500. Comp Biochem Physiol Part D Genomics Proteomics.

[54] Huang, Z. X. Transcriptomic analysis on early developmental stages of *Haliotis diversicolor*. Master’s degree thesis of Xiamen university 2012.

[55] Lopaschuk GD, Spafford MA, Marsh DR (1991). Glycolysis is predominant source of myocardial ATP production immediately after birth. Am J Physiol.

[56] Heras H, Garin CF, Pollero RJ (1998). Biochemical composition and energy sources during embryo development and in early juveniles of the snail *Pomacea canaliculata* (Mollusca: Gastropoda). J Exp Zool.

[57] De DC, Wattiaux R (1966). Functions of lysosomes. Annu Rev Physiol.

[58] Werner GDA, Gemmell P, Grosser S, Hamer R, Shimeld SM (2013). Analysis of a deep transcriptome from the mantle tissue of *Patella vulgata* Linnaeus (Mollusca: Gastropoda: Patellidae) reveals candidate biomineralising genes. Mar Biotechnol.

[59] Chukwuka CO (2014). Eco-physiological adaptation of the land snail *Achatina achatina* (Gastropoda: Pulmonata) in tropical agro-ecosystem. Journal of Basic and Applied Zoology.

[60] Chen CS, Alonso JL, Ostuni E, Whitesides GM, Ingber DE (2003). Cell shape provides global control of focal adhesion assembly. Biochem Bioph Res Co.

[61] Zamir E, Geiger B (2001). Components of cell-matrix adhesions. J Cell Sci.

[62] Alldinger S (2006). Roles of an extracellular matrix (ECM) receptor and ECM processing enzymes in demyelinating canine distemper encephalitis. Dtsch Tierarztl Wochenschr.

[63] Shi YB, Fu L, Hasebe T, Ishizuya-Oka A (2007). Regulation of extracellular matrix remodeling and cell fate determination by matrix metalloproteinase stromelysin-3 during thyroid hormone-dependent postembryonic development. Pharmacol Ther.

[64] Fujimoto K, Nakajima K, Yaoita Y (2007). Expression of matrix metalloproteinase genes in regressing or remodeling organs during amphibian metamorphosis. Dev Growth Differ.

[65] Royer V, Hourdry A, Fraichard S, Bouhin H (2004). Characterization of a putative extracellular matrix protein from the beetle *Tenebrio molitor*: hormonal regulation during metamorphosis. Dev Genes Evol.

[66] Halet G, Viard P, Carroll J (2008). Constitutive PtdIns (3, 4, 5) P3 synthesis promotes the development and survival of early mammalian embryos. Development.

[67] Yin MM (2014). Progress on PI3k/Akt signaling pathway regulating self-renewal and pluripotency of embryonic stem cells. Sheng Li Xue Bao (in Chinese).

[68] Cleveland DW (1980). Number and evolutionary conservation of alpha- and beta-tubulin and cytoplasmic beta- and gamma-actin genes using specific cloned cDNA probes. Cell.

[69] Ignotz RA, Massagué J (1987). Cell adhesion protein receptors as targets for transforming growth factor-β action. Cell.

[70] Karsi A, Patterson A, Feng J, Liu Z (2002). Translational machinery of channel catfish: I. A transcriptomic approach to the analysis of 32 40S ribosomal protein genes and their expression. Gene.

[71] Labban M, Sossin WS (2011). Translation of 5′ terminal oligopyrimidine tract (5′TOP) mRNAs in *Aplysia californica* is regulated by the target of rapamycin (TOR). Biochem Bioph Res Co.

[72] Mann K, Edsinger E (2014). The *Lottia gigantea* shell matrix proteome: re-analysis including MaxQuant iBAQ quantitation and phosphoproteome analysis. Proteome Sci.

[73] Juancarlos SH, Juliohumberto CM (2009). Activity of trypsin from *Litopenaeus vannamei*. Aquaculture.

[74] Degnan BM, Groppe JC, Morse DE (1995). Chymotrypsinm RNA expression in digestive gland amoebocytes: cell specification occurs prior tometamorphosis and gut morphogenesis in the gastropod,*Haliotis rufescens*. Roux’s Arch Dev BioI.

[75] Agrawal VP, Sastry KV, Kaushab SK (1975). Digestive enzymes of three teleost fishes. Acta Physiol Acad Sci Hung.

[76] Davidson B, Swalla BJ (2002). A molecular analysis of ascidian metamorphosis reveals activation of an innate immune response. Development.

[77] Wang XW, Wang JX (2013). Diversity and multiple functions of lectins in shrimp immunity. Dev Comp Immunol.

[78] Bao XB (2015). A C-type lectin fold gene from Japanese scallop *Mizuhopecten yessoensis*, involved with immunity and metamorphosis. Genet Mol Res.

[79] Gunter HM, Degnan BM (2007). Developmental expression of Hsp90, Hsp70 and HSF during morphogenesis in the vetigastropod *Haliotis asinina*. Dev Genes Evol.

[80] Searcybernal R, Anguianobeltran C (1998). Optimizing the concentration of gamma-aminobutyric acid (GABA) for inducing larval metamorphosis in the red abalone *Haliotis rufescens* (Mollusca: Gastropoda). J World Aquacult Soc.

[81] Fang J (1999). Inducement of settlement and metamorphosis by chemical cues in *Tegillarca granosalarvae*. Journal of Fishery Sciences of China.

[82] Ke C (2001). Induction of settlement in Japanese abalone, *Haliotis discus discus*. Journal of Oceanography in Taiwan Strait.

[83] Morse DE, Hooker N, Jensen L (1979). C-aminobutyric acid, a neurotransmitter, induces planktonic abalone larvae to settle and begin metamorphosis. Science.

[84] Garcíalavandeira M (2005). Effects of GABA and epinephrine on the settlement and metamorphosis of the larvae of four species of bivalve molluscs. J Exp Mar Biol Ecol.

[85] Woods RG (2004). Gene expression during early ascidian metamorphosis requires signalling by Hemps, an EGF-like protein. Development.

[86] Roberts B (2007). A complement response may activate metamorphosis in the ascidian *Boltenia villosa*. Dev Genes Evol.

[87] Grasso LC (2008). Microarray analysis identifies candidate genes for key roles in coral development. BMC Genomics.

[88] Maki JS, Mitchell R (1985). Involvement of lectins in the settlement and metamorphosis of marine invertebrate larvae. B Mar Sci.

[89] Matsutani T, Morishita K, Seki T, Mori K (2001). Involvement of lectin-like factors in larval settlement and metamorphosis in the abalone, *Haliotis discus hannai*. Tohoku Journal of Agricultural Research.

[90] Jackson DJ, Wö rheide W, Degnan BM (2007). Dynamic expression of ancient and novel molluscan shell genes during ecological transitions. BMC Evol Biol.

[91] Chung HJ, Sehnke PC, Ferl RJ (1999). The 14-3-3 proteins: cellular regulators of plant metabolism. Trends Plant Sci.

[92] Feng L (2004). PKA phosphorylation and 14-3-3 interaction regulate the function of neurofibromatosis type 1 tumor suppressor neurofibromin. FEBS Lett.

[93] Oksvold MP, Huitfeldt HS, Langdon WY (2004). Identification of 14-3-3 zeta as an EGF receptor interacting protein. FEBS Lett.

[94] Skoulakis EMC, Davis RL (1998). 14-3-3 proteins in neuronal development and function. Mol Neurobiol.

[95] Toyo-Oka K (2003). 14-3-3 is important for neuronal migration by binding to nudel, a molecular explanation for miller-dieker syndrome. Nature Genet.

[96] Bunney T, De Boer A, Levin M (2003). Fusicoccin signaling reveals 14-3-3 protein function as a novel step in left-right patterning during amphibian embryogenesis. Development.

[97] Vera A (2013). Sco-spondin from embryonic cerebrospinal fluid is required for neurogenesis during early brain development. Aquac Res.

[98] Miao Y (2015). RNA sequencing identifies upregulated kyphoscoliosis peptidase and phosphatidic acid signaling pathways in muscle hypertrophy generated by transgenic expression of myostatin propeptide. Int J Mol Sci.

[99] Götz S (2008). High-throughput functional annotation and data mining with the Blast2GO suite. Nucleic Acids Res.

[100] Ogata H, Goto S, Fujibuchi W, Kanehisa M (1998). Computation with the KEGG pathway database. Biosystems.

