# Author Correction: Proteomics Studies on the three Larval Stages of Development and Metamorphosis of *Babylonia areolata*

**DOI:** 10.1038/s41598-018-28807-x

**Published:** 2018-07-23

**Authors:** Minghui Shen, Guilan Di, Min Li, Jingqiang Fu, Qi Dai, Xiulian Miao, Miaoqin Huang, Weiwei You, Caihuan Ke

**Affiliations:** 1 State Key Laboratory of Marine Environmental Science, College of Ocean and Earth Sciences, Xiamen University, Xiamen, 361005 China; 2 College of Fisheries, Henan Normal University, Xinxiang, 453007 China; 3 Hainan Academy of Ocean and Fisheries Sciences, Haikou, 570206 China; 4 College of Life Sciences, Liaocheng University, Liaocheng, 252059 China

Correction to: *Scientific Reports* 10.1038/s41598-018-24645-z, published online 19 April 2018

The original version of this Article contained errors in the Affiliations.

Minghui Shen was incorrectly affiliated with ‘College of Fisheries, Henan Normal University, Xinxiang, 453007, China’, and an additional affiliation was omitted. The correct affiliations are listed below:

State Key Laboratory of Marine Environmental Science, College of Ocean and Earth Sciences, Xiamen University, Xiamen, 361005, China

Hainan Academy of Ocean and Fisheries Sciences, Haikou, 570206, China

In addition, Min Li, Jingqiang Fu, Qi Dai, Miaoqin Huang, Weiwei You and Caihuan Ke were incorrectly affiliated with ‘College of Fisheries, Henan Normal University, Xinxiang, 453007, China’.

Finally, Guilan Di was incorrectly affiliated with ‘Hainan Academy of Ocean and Fisheries Sciences, Haikou, 570206 China’.

These errors have now been corrected in the PDF and HTML versions of the Article.

