# Supplementary material for: Proteomics Studies on the three Larval Stages of Development and Metamorphosis of Babylonia areolata
Source: Sci Rep. 2018 Apr 19;8:6269. doi: 10.1038/s41598-018-24645-z (PMC5908917; doi:10.1038/s41598-018-24645-z)
Supplement: Supplementary file 1 — Supplementary figures and tables [file 41598_2018_24645_MOESM1_ESM.pdf]

Proteomics Studies on the three Larval Stages of Development and Metamorphosis of *Babylonia areolata*

Minghui Shen[1,3#],Guilan Di[1, 2#]*, Min Li[1], Jingqiang Fu[1], Qi Dai[1], Xiulian Miao[4], Miaoqin Huang[1], Weiwei You[1], Caihuan Ke[1]*

[1]State Key Laboratory of Marine Environmental Science, Xiamen University, Xiamen, China; College of Ocean and Earth Sciences, Xiamen University, Xiamen, China

[2]College of Fisheries, Henan Normal University, Xinxiang, 453007, China

[3]Hainan Academy of Ocean and Fisheries Sciences, Haikou, 570206, China

[4]College of Life Sciences, Liaocheng University, Liaocheng, 252059, China

[#] These authors contributed equally to this work

* Co-corresponding author: Guilan Di, College of Fisheries, Henan Normal University, Xinxiang, 453007, China, Tel: ＋86 3733326563 E-mail: gldi123@163.com; Corresponding author: Cai-huan Ke, State Key Laboratory of Marine Environmental Science, Xiamen University, Fujian Province 361005, PR China, Tel: ＋86 592 2187420 E-mail: chke@xmu.edu.cn;

Running title: Proteomics of *Babylonia areolata* larvae

Titles and legends of figures

Supplementary Fig. 1 Significantly enriched KEGG pathway (Ribosome)

Supplementary Fig. 2 Significantly enriched KEGG pathway (Carbon metabolism)

Supplementary Fig. 3 Significantly enriched KEGG pathway (Lysosome)

Supplementary Fig. 4 KEGG pathways related with the early development (PI3K-AKT)

Supplementary Fig. 5 KEGG pathways related with the early development (ERBB)

Supplementary Fig. 6 KEGG pathways related with the early development (mTOR)

Supplementary Fig.7 KEGG pathways related with the early development (Glycolysis /Gluconeogenesis)

Supplementary Fig. 8 KEGG pathways related with the early development (Biosynthesis of amino acids)

Supplementary Fig. 9 KEGG pathways related with the early development (Protein digestion and absorption)

Supplementary Fig. 10 KEGG pathways related with the early development (Focal adhesion)

Supplementary Fig. 11 KEGG pathways related with the early development (GABAergic synapse)

Supplementary Fig. 12 KEGG pathways related with the early development (Phagosome)

Supplementary Figure 1

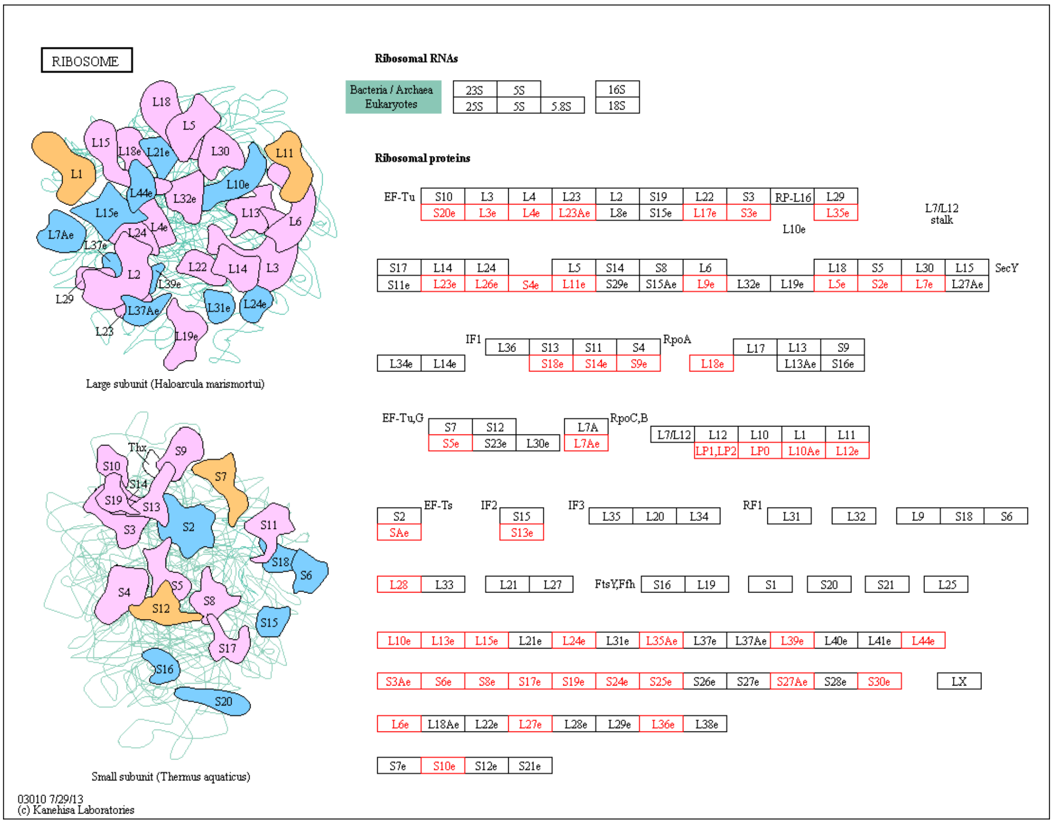

Supplementary Figure 2

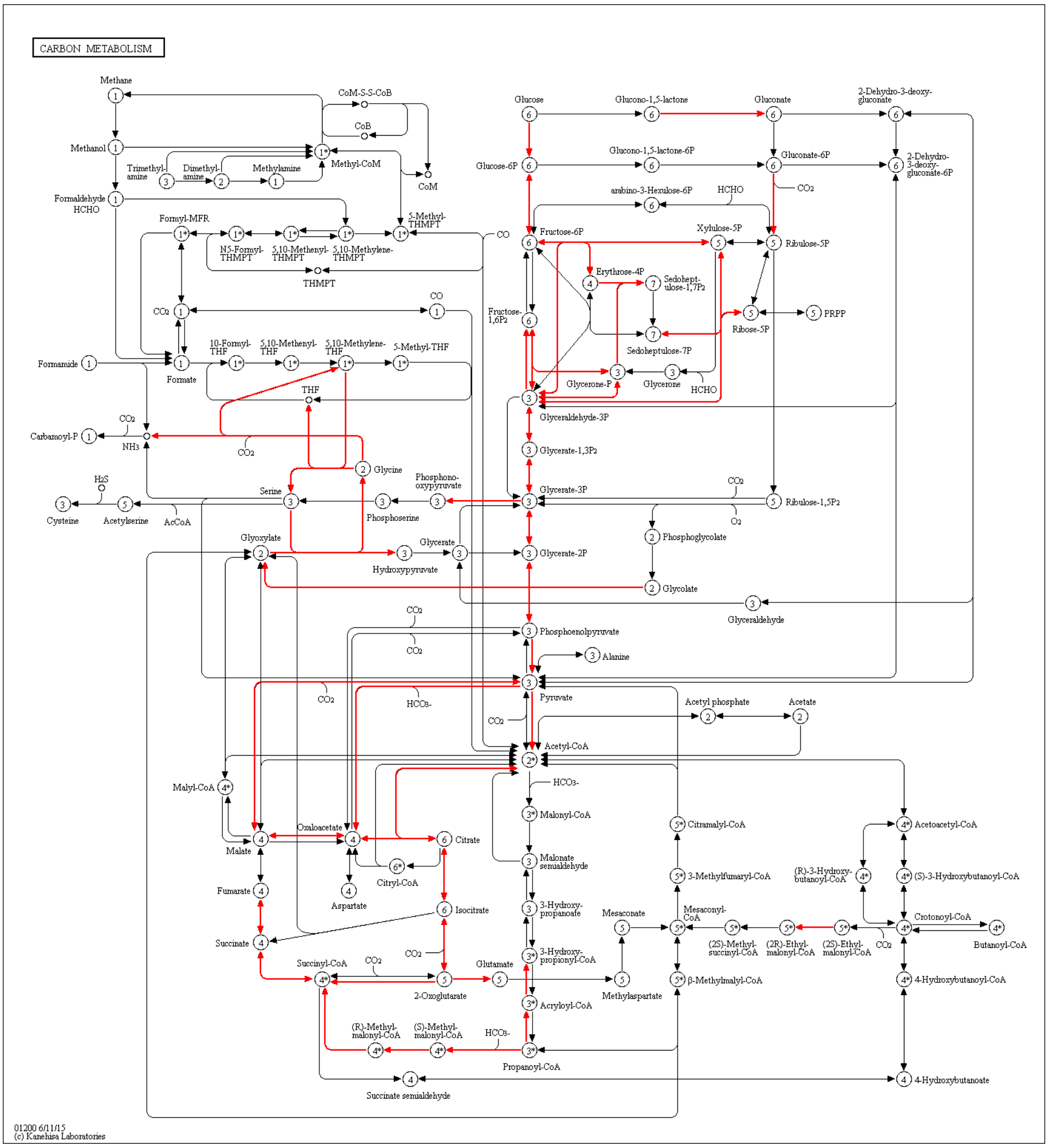

Supplementary Figure 3

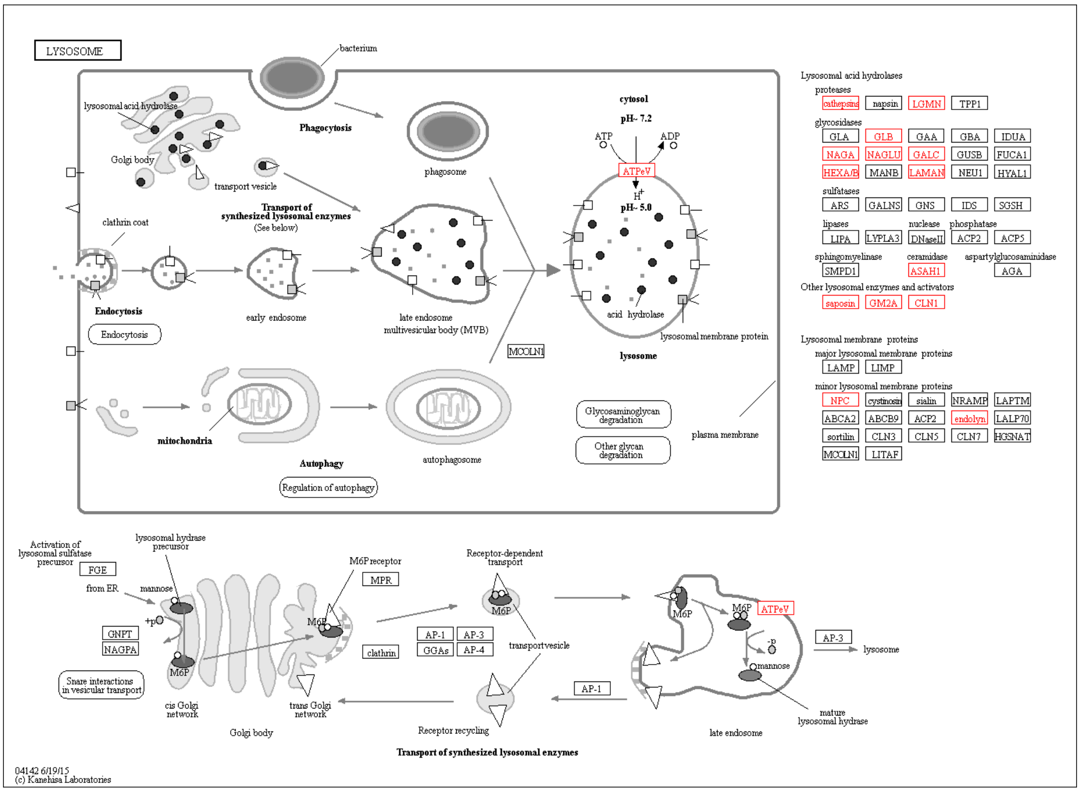

Supplementary Figure 4

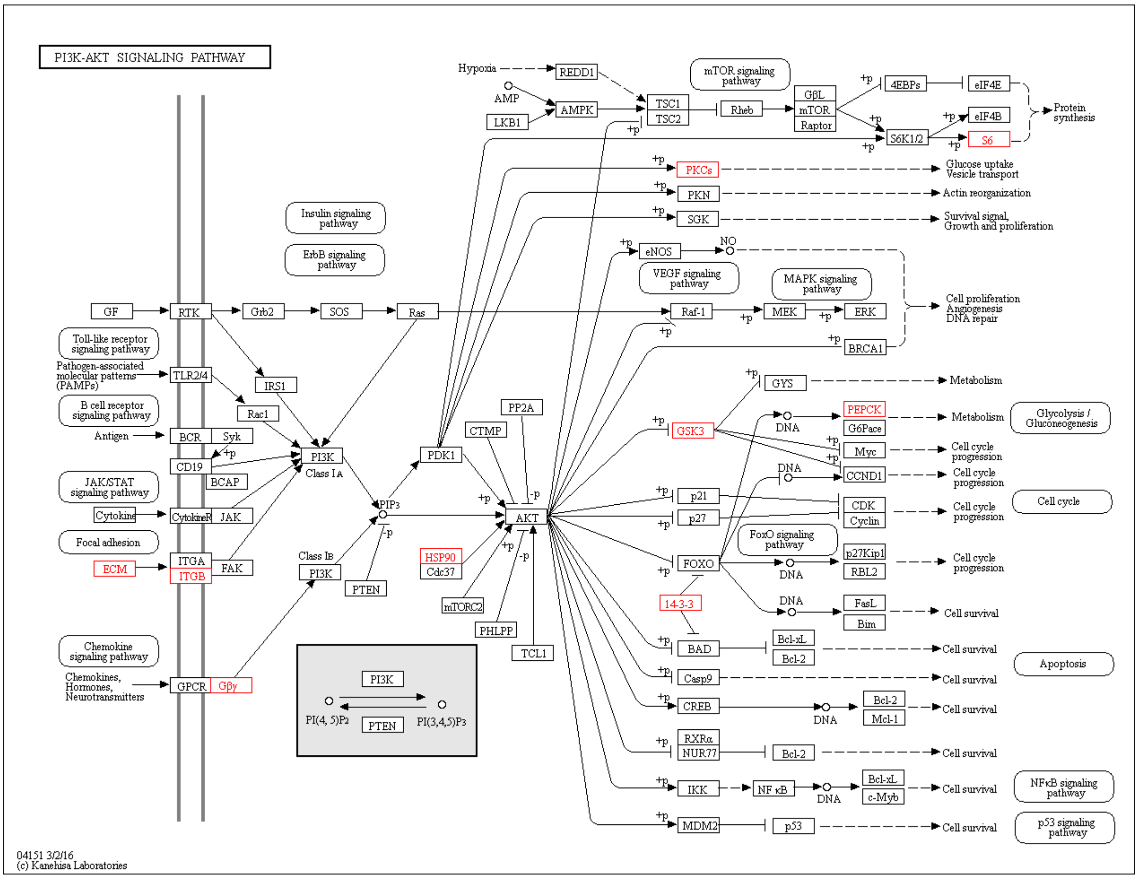

Supplementary Figure 5

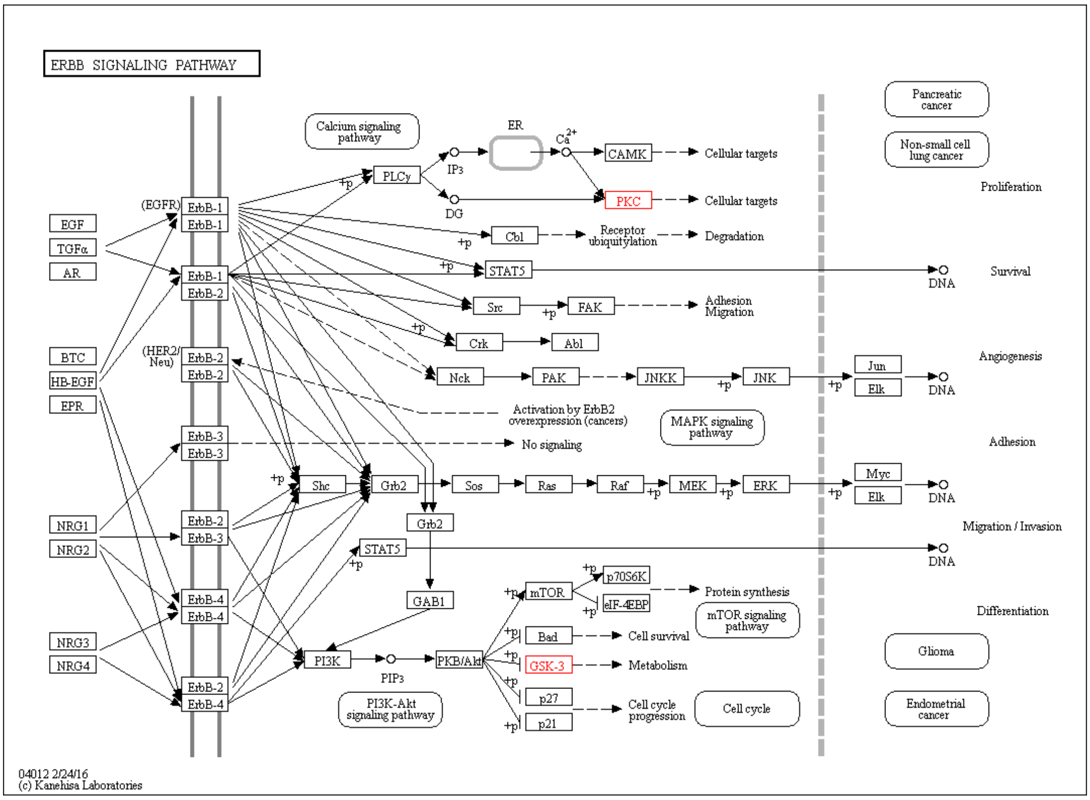

Supplementary Figure 6

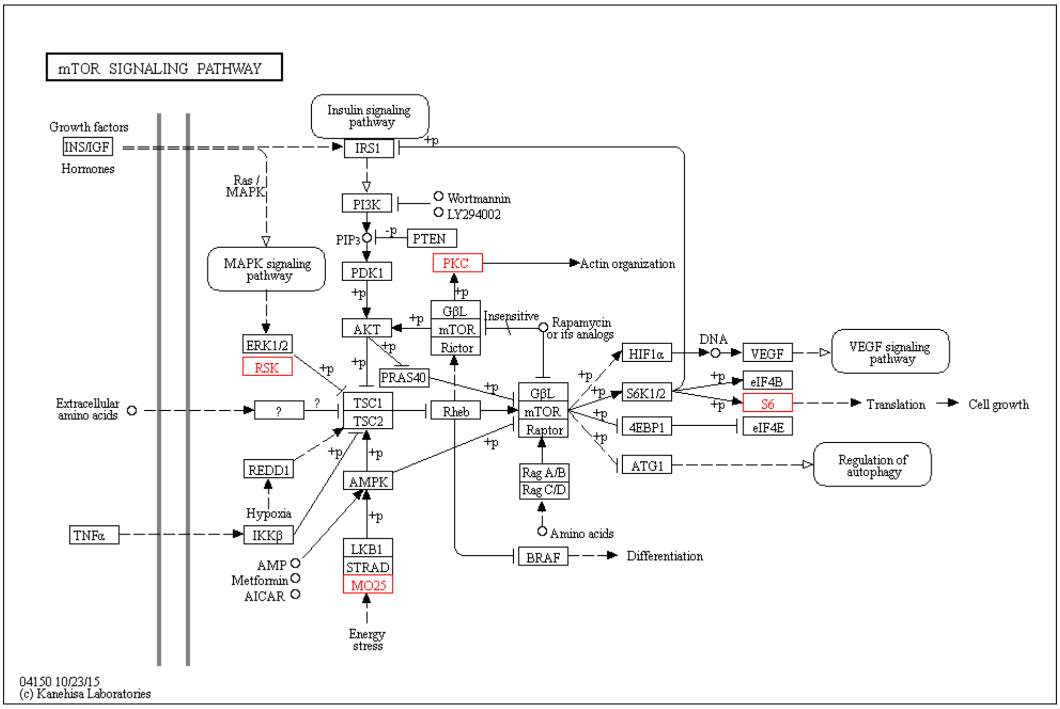

Supplementary Figure 7

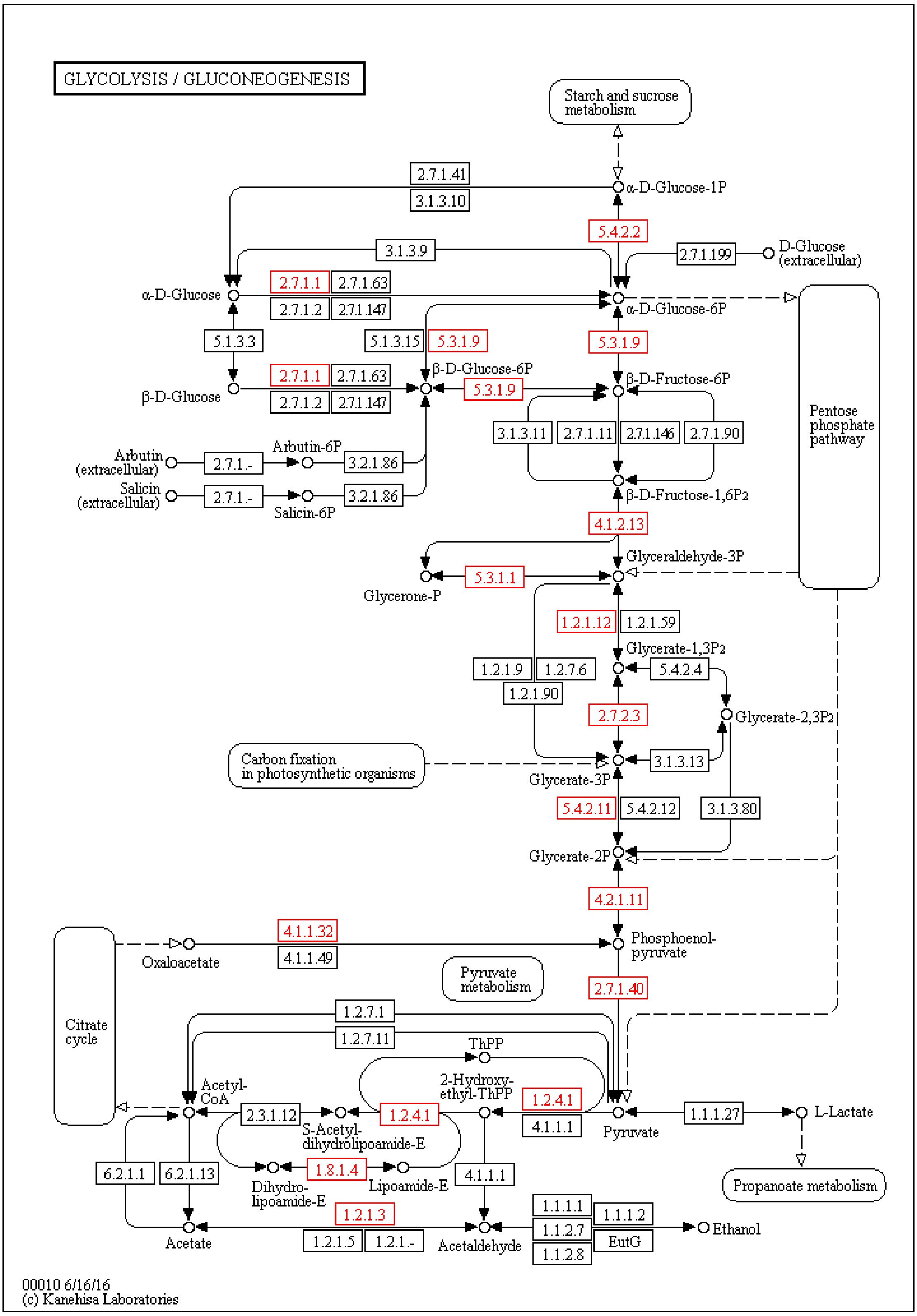

Supplementary Figure 8

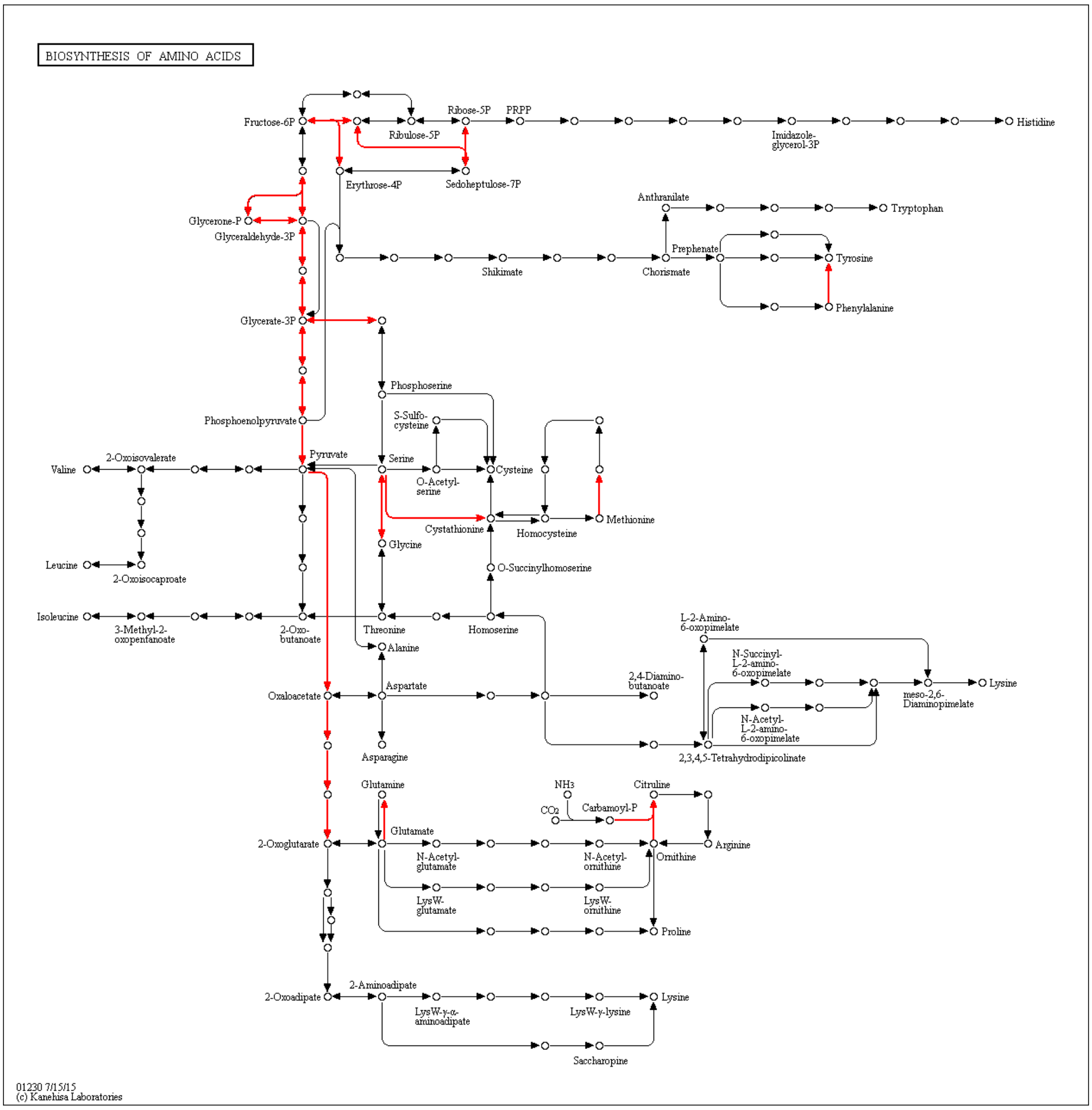

Supplementary Figure 9

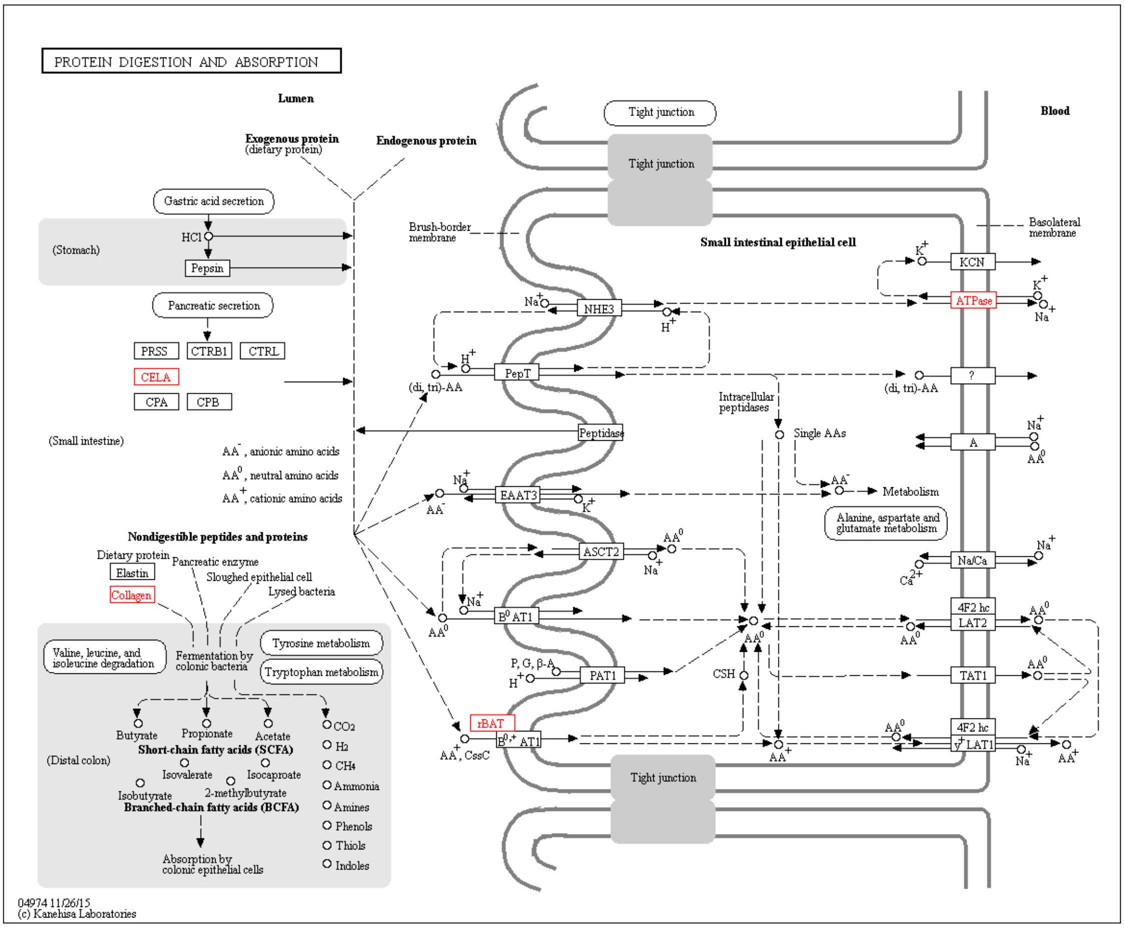

Supplementary Figure 10

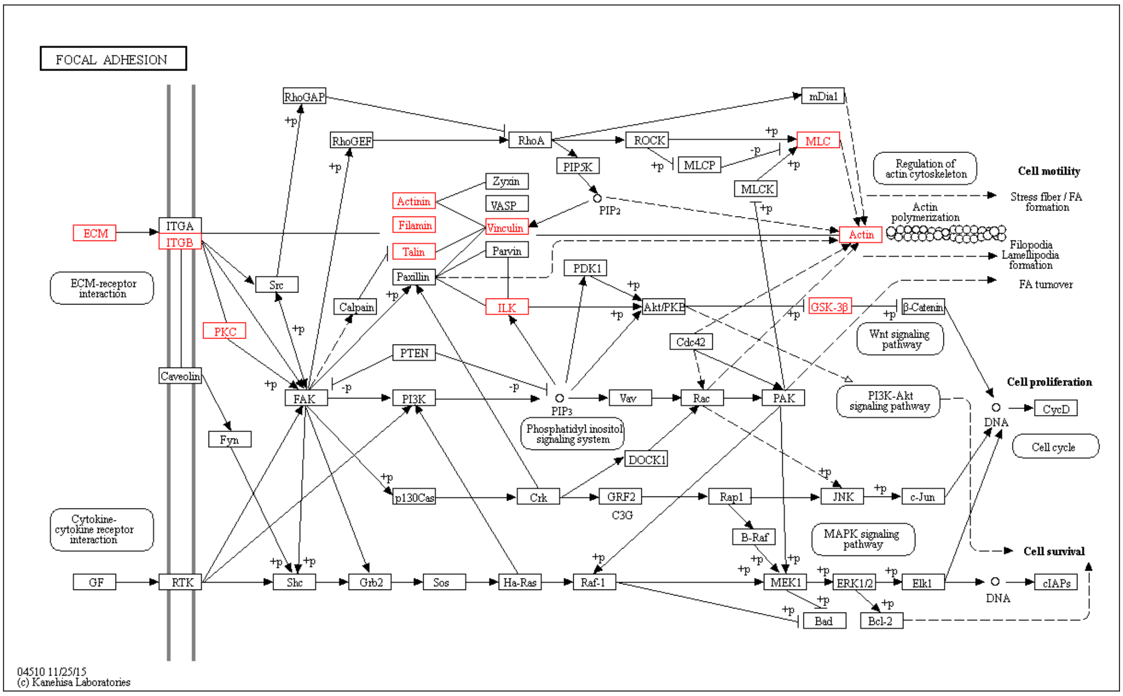

Supplementary Figure 11

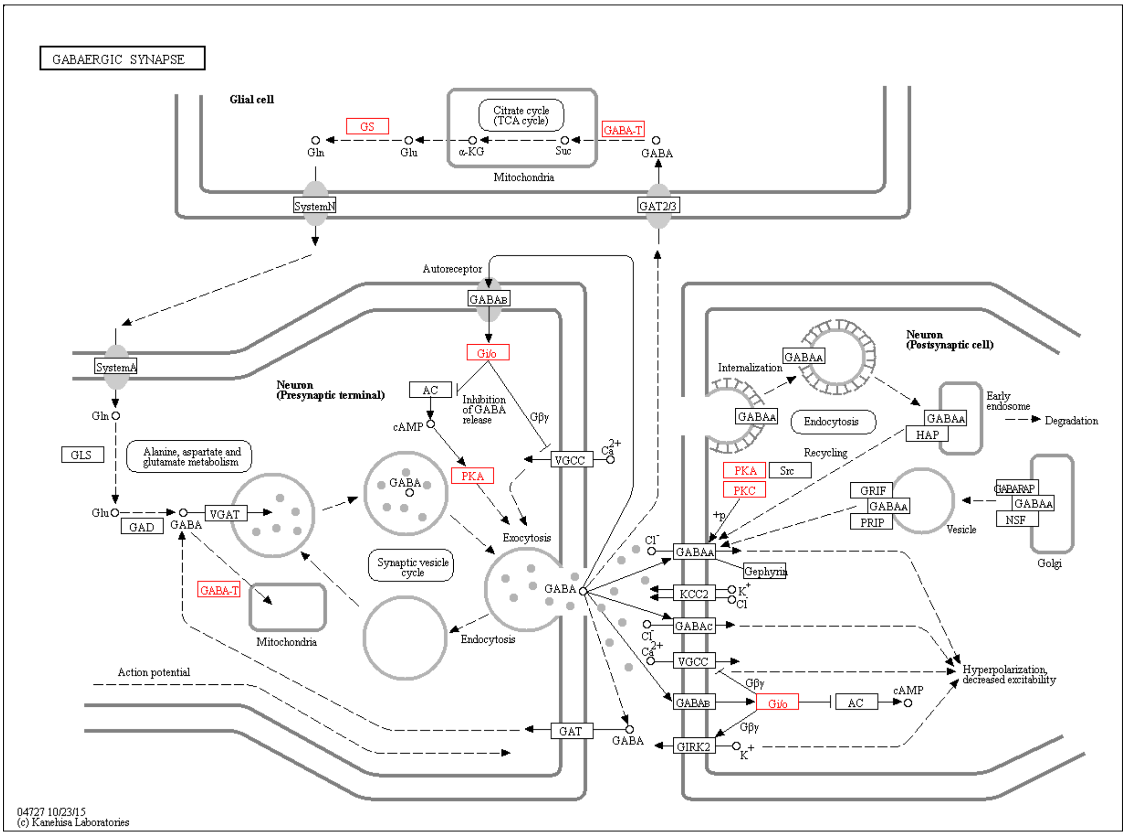

Supplementary Figure 12

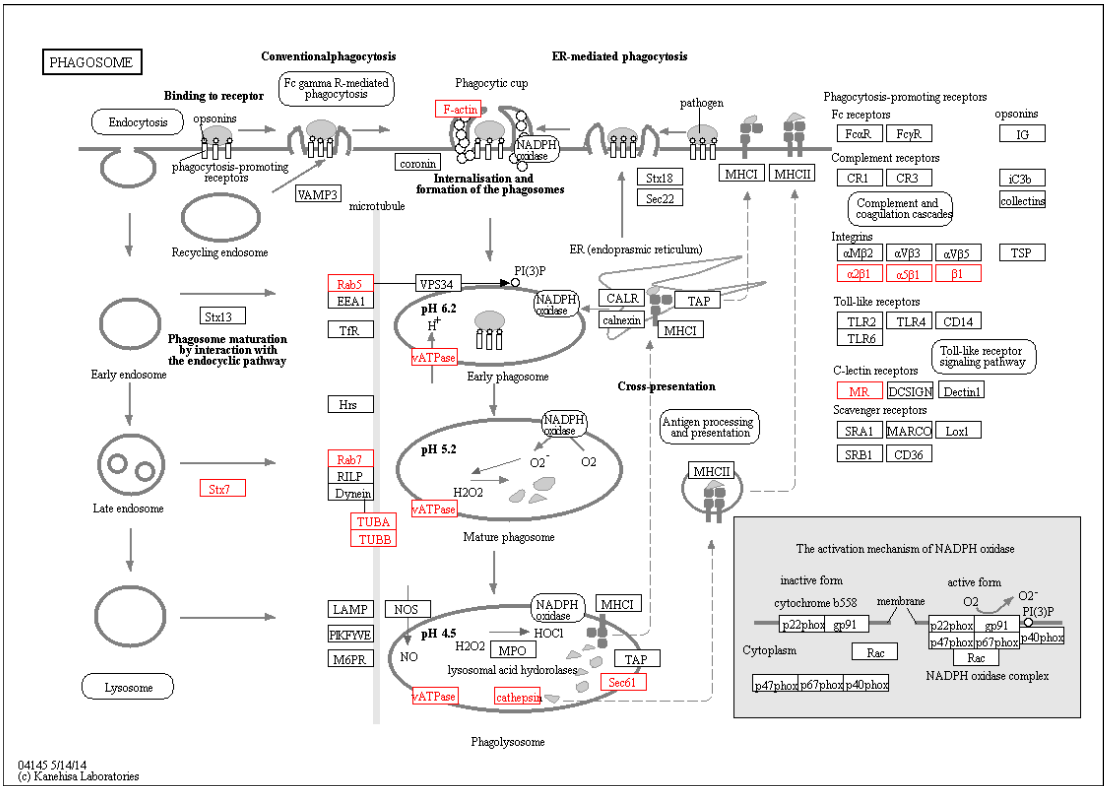

Supplementary Table 1 Differential proteins with important physiological functions between ZRZ_V and ZRZ_III with protein volumes calculated

NO	Protein IDs	Description	Proteins	Unique peptides	Sequence coverage [%]	Mol. weight [kDa]	average ZRZ_III	average ZRZ_V	ZRZ_V/ZRZ_III	t test p value
1	>c258005_g1	nidogen-2-like [Crassostrea gigas]	1	5	41.4	26.963	236466666.7	109230000	0.461926	0.000725
2	>c268605_g3	fibrinolytic enzyme, isozyme C-like [Biomphalaria glabrata]	1	5	33.5	24.863	126666666.7	54475666.67	0.430071	0.006406
3	>c260447_g1	alanine--glyoxylate aminotransferase 2, mitochondrial-like [Aplysia californica]	1	4	13.5	32.278	307640000	131430000	0.42722	2.12E-05
4	>c262433_g1	EF-hand calcium-binding domain-containing protein 1-like isoform X1 [Aplysia californica]	1	6	32.1	24.276	106922666.7	43814333.33	0.409776	0.000571
5	>c267093_g5	radial spoke head 1 homolog [Aplysia californica]	1	1	31.8	9.7533	78818500	23769000	0.301566	0.006267
6	>c191430_g1	calcium-binding mitochondrial carrier protein SCaMC-2-like [Biomphalaria glabrata]	1	1	9.8	10.007	964250000	204846666.7	0.212441	0.002092
7	>c241598_g1	Calmodulin [Fusarium oxysporum f. sp. cubense race 1]	1	1	10.4	21.745	13150000			
8	>c260959_g3	BTB/POZ domain-containing protein 16-like isoform X2 [Aplysia californica]	1	3	5.2	75.898	17258333.33			
9	>c174971_g2	glycogen [starch] synthase-like [Aplysia californica]	1	1	11.1	17.25		10895300		
10	>c242440_g3;>c242440_g4;>c408621_g1;>c383033_g1;>c113549_g1;>c159761_g1	14-3-3 protein [Penaeus monodon]	6	1	23.1	27.825		154226666.7		

11	>c258421_g1	SCO-spondin-like [Biomphalaria glabrata]	1	4	4.8	88.238	124433333.3
12	>c261121_g1	marginal zone B- and B1-cell-specific protein-like [Saccoglossus kowalevskii]	1	2	30.2	7.198	101944666.7
13	>c264589_g1	chitinase-like lectin [Littorina littorea]	1	4	9.8	59.333	32027333.33
14	>c267235_g3	intraflagellar transport protein 140 homolog [Aplysia californica]	1	1	6.9	12.418	733436666.7
15	>c408963_g1	kinesin-like protein KIF28P, partial [Biomphalaria glabrata]	1	1	10	26.682	14733333.33
16	>c442420_g1	Lethal(2) giant larvae-like protein 1 [Crassostrea gigas]	1	3	55.6	8.1788	40973666.67

Supplementary Table 2 Differential proteins with important physiological functions between ZRZ_VI and ZRZ_III with protein volumes calculated

NO	Protein IDs	Description	Proteins	Unique peptides	Sequence coverage [%]	Mol. weight [kDa]	average ZRZ_III	average ZRZ_VI	ZRZ_VI/ZRZ_III	t test p value
1	>c255545_g2	nidogen-2-like [Crassostrea gigas]	1	8	41.9	32.201	77810500	1.98E+09	25.44687	0.036717
2	>c267414_g2	cathepsin L2, partial [Reishia clavigera]	1	8	41.3	42.199	1.75E+08	2.23E+09	12.73731	0.000103
3	>c266601_g4	carboxypeptidase B-like [Crassostrea gigas]	1	12	30.4	51.489	34411000	4.31E+08	12.52807	0.020682
4	>c258308_g1	chymotrypsin-like protease CTRL-1 isoform X1 [Mandrillus leucophaeus]	1	9	46.9	28.635	75114000	8.64E+08	11.50474	0.000213
5	>c254358_g1	universal stress protein A-like protein [Aplysia californica]	1	3	39.6	11.423	61341500	2.93E+08	4.772408	0.01742
6	>c261202_g2	cathepsin C [Sinonovacula constricta]	1	9	29.7	51.868	2.6E+08	1.03E+09	3.980271	7.04E-06
7	>c263412_g1	cartilage matrix protein-like [Biomphalaria glabrata]	1	21	61.1	48.168	8.03E+08	3.13E+09	3.902029	0.000567
8	>c435748_g1	kyphoscoliosis peptidase-like [Crassostrea gigas]	1	7	45.5	21.999	93662667	3.42E+08	3.649409	0.001868
9	>c252747_g1	cellulase EGX3 [Pomacea canaliculata]	1	5	32.5	26.694	1.88E+08	36127000	0.191883	0.003492
10	>c265114_g2	C-type lectin [Littorina littorea]	1	7	14.6	74.245	2.22E+08	36884000	0.166462	0.000725
11	>c232056_g1	cubilin-like [Crassostrea gigas]	1	7	62.2	18.544	1.64E+09	2.66E+08	0.162162	9.91E-06
12	>c191430_g1	calcium-binding mitochondrial carrier protein SCaMC-2-like [Biomphalaria glabrata]	1	1	9.8	10.007	9.64E+08	1.34E+08	0.139284	0.001853
13	>c249098_g1;>c238726_g2	testis, prostate and placenta-expressed protein-like isoform X1 [Lingula anatina]	2	8	37.5	25.812	3.03E+08	34210500	0.112836	0.011434
14	>c265341_g2;REV__>c265125_g1;>c331230_g1	rootletin-like isoform X6 [Crassostrea gigas]	3	94	50.3	243.83	1.04E+10	1.04E+09	0.100048	2.28E-07
15	>c250843_g2	tektin-3-like isoform X3 [Crassostrea gigas]	1	7	61	13.594	1.81E+09	1.69E+08	0.093436	0.000949

16	>c258546_g1	alpha amylase 1 [Haliotis discus discus]	1	17	50.2	56.904	1.01E+09	84317667	0.083082	4.65E-05
17	>c262177_g1	tektin-3-like [Aplysia californica]	1	17	56.7	40.272	5.1E+09	2.02E+08	0.039576	7.29E-06
18	>c205639_g1	serine protease CFSP2 [Azumapecten farreri]	1	3	14.9	36.536	41811333			
19	>c217572_g1	amyloid protein-binding protein 2-like isoform X2 [Aplysia californica]	1	4	13.4	33.581	18455333			
20	>c235707_g1	intraflagellar transport protein 46 homolog [Aplysia californica]	1	1	3.9	28.945	9181300			
21	>c258005_g1	nidogen-2-like [Crassostrea gigas]	1	5	41.4	26.963	2.36E+08			
22	>c259006_g2	Calpain-5 [Crassostrea gigas]	1	6	12.4	76.196	30695333			
23	>c260145_g6	probable glutathione S-transferase 7 [Biomphalaria glabrata]	1	2	18	24.455	48318333			
24	>c260959_g3	BTB/POZ domain-containing protein 16-like isoform X2 [Aplysia californica]	1	3	5.2	75.898	17258333			
25	>c261187_g1	G patch domain-containing protein 2-like [Crassostrea gigas]	1	6	54.1	16.049	64754667			
26	>c261220_g1	carboxypeptidase D-like [Biomphalaria glabrata]	1	1	0.8	142.39	9011033			
27	>c262433_g1	EF-hand calcium-binding domain-containing protein 1-like isoform X1 [Aplysia californica]	1	6	32.1	24.276	1.07E+08			
28	>c264276_g1	pancreatic lipase-related protein 2-like [Crassostrea gigas]	1	1	2.3	55.628	14677000			
29	>c266550_g5	EF-hand calcium-binding domain-containing protein 5-like [Aplysia californica]	1	4	9.9	59.797	35338667			
30	>c267924_g1	intraflagellar transport protein 56-like [Aplysia californica]	1	2	5.6	64.285	26018000			
31	>c223366_g1	C-type lectin domain family 10 member A-like	1	1	9.3	14.233		21688000		

		[Poecilia mexicana]					
32	>c245773_g1	lysosomal Pro-X carboxypeptidase-like [Lingula anatina]	1	3	15.4	20.601	27021000
33	>c253650_g3	integrin alpha-8-like [Aplysia californica]	1	3	12.2	31.989	47606000
34	>c257164_g3	transmembrane protease serine 3-like [Metaseiulus occidentalis]	1	6	30.6	26.17	1.51E+08
35	>c257469_g1	von Willebrand factor D and EGF domain-containing protein, partial [Strongylocentrotus purpuratus]	1	1	5.4	22.765	13194133
36	>c258421_g1	gi|908445639|SCO-spondin-like [Biomphalaria glabrata]	1	4	4.8	88.238	80159333
37	>c261121_g1	marginal zone B- and B1-cell-specific protein-like [Saccoglossus kowalevskii]	1	2	30.2	7.198	1.18E+08
38	>c264589_g1	gi|735659563|chitinase-like lectin [Littorina littorea]	1	4	9.8	59.333	63295667
39	>c265793_g2	gi|908448079|antitrypsin-like isoform X2 [Biomphalaria glabrata]	1	3	9.5	40.517	71065667
40	>c265793_g4	gi|312190476|serine protease inhibitor protein [Haliotis discus discus]	1	2	13.8	17.93	17203667
41	>c267235_g3	intraflagellar transport protein 140 homolog [Aplysia californica]	1	1	6.9	12.418	1.35E+08
42	>c267471_g2	gi|82698283serine protease CFSP2 [Azumapecten farreri]	1	5	22.7	30.321	1.54E+08
43	>c267791_g1	gi|325197123|carboxypeptidase E-3 precursor [Aplysia californica]	1	1	8.2	28.278	18088333
44	>c368924_g1	gi|724970559|heat shock 70 kDa protein cognate 4-like, partial [Rhinopithecus roxellana]	1	2	50	9.866	49336667
	>c442420_g1	gi|405952027|Lethal(2) giant larvae-like protein 1	1	3	55.6	8.1788	40422333

45	[Crassostrea gigas]

Supplementary Table 3 Differential proteins with important physiological functions between ZRZ_VI and ZRZ_V with protein volumes calculated

NO	Protein IDs	Description	Proteins	Unique peptides	Sequence coverage [%]	Mol. weight [kDa]	average ZRZ_V	average ZRZ_VI	ZRZ_VI/ ZRZ_V	t test p value
1	>c255545_g2	gi|762081924|nidogen-2-like [Crassostrea gigas]	1	8	41.9	32.201	60406000	1.98E+09	32.77875	0.009332
2	>c267414_g2	gi|356984263|cathepsin L2, partial [Reishia clavigera]	1	8	41.3	42.199	1.26E+08	2.23E+09	17.70006	0.000108
3	>c256163_g2	gi|356984263|cathepsin L2, partial [Reishia clavigera]	1	5	28.9	24.831	23603333	2.31E+08	9.806666	0.004756
4	>c258308_g1	gi|795233655|chymotrypsin-like protease CTRL-1 isoform X1 [Mandrillus leucophaeus]	1	9	46.9	28.635	92125000	8.64E+08	9.380371	3.37E-05
5	>c254358_g1	gi|524874093|universal stress protein A-like protein [Aplysia californica]	1	3	39.6	11.423	91542333	2.93E+08	3.197938	0.006334
6	>c252747_g1	gi|113473655|cellulase EGX3 [Pomacea canaliculata]	1	5	32.5	26.694	1.77E+08	36127000	0.203712	0.000454
7	>c265114_g2	gi|735659613|C-type lectin [Littorina littorea]	1	7	14.6	74.245	2.07E+08	36884000	0.177837	0.000189
8	>c265341_g2; REV__>c265125_g1;>c331230_g1	gi|762100293|rootletin-like isoform X6 [Crassostrea gigas]	3	94	50.3	243.83	7.04E+09	1.04E+09	0.148378	8.79E-08
9	>c266019_g6	gi|523582279|goose-type lysozyme [Haliotis discus discus]	1	6	37.8	30.104	4.08E+08	58950000	0.144517	0.000204
10	>c255609_g2	gi|762092090|periostin-like [Crassostrea gigas]	1	3	19.7	32.685	3.01E+08	41502667	0.137672	0.000103

11	>c258546_g1	gi|126697306|alpha amylase 1 [Haliotis discus discus]	1	17	50.2	56.904	6.75E+08	84317667	0.124875	3.77E-05
12	>c259771_g1	gi|871225116|cilia- and flagella-associated protein 45-like [Aplysia californica]	1	18	33.5	66.189	4.74E+08	51362667	0.108457	7.82E-05
13	>c257841_g1	gi|908450794|rootletin-like [Biomphalaria glabrata]	1	15	25.9	105.65	4.08E+08	42516000	0.104299	0.001094
14	>c262177_g1	gi|871271530|tektin-3-like [Aplysia californica]	1	17	56.7	40.272	2.55E+09	2.02E+08	0.07916	1.76E-05
15	>c262177_g2	gi|908443323|tektin-3-like isoform X2 [Biomphalaria glabrata]	1	1	21.1	14.656	4.36E+08	25906667	0.059434	0.001986
16	>c199415_g1	gi|908442840|EF-hand calcium-binding domain-containing protein 1-like [Biomphalaria glabrata]	1	2	11.7	31.171	15685333			
17	>c235707_g1	gi|871240026|intraflagellar transport protein 46 homolog [Aplysia californica]	1	1	3.9	28.945	8700700			
18	>c240030_g3	gi|524901544|ubiquitin fusion degradation protein 1 homolog isoform X2 [Aplysia californica]	1	2	8.6	36.91	12120333			
19	>c241050_g1	gi|762100289|rootletin-like isoform X4 [Crassostrea gigas]	1	4	16.7	40.585	35504333			
20	>c242440_g3; >c242440_g4; >c408621_g1; >c383033_g1; >c113549_g1; >c159761_g1	gi|330690205|14-3-3 protein [Penaeus monodon]	6	1	23.1	27.825	1.54E+08			
	>c243065_g1	gi|735659663|C-type lectin [Littorina littorea]	1	4	7.4	59.398	71213000			

21								
22	>c257900_g1	gi|871270388| rootletin-like [Aplysia californica]	1	1	19.1	12.737	32262000	
23	>c258005_g1	gi|762081924| nidogen-2-like [Crassostrea gigas]	1	5	41.4	26.963	1.09E+08	
24	>c260145_g6	gi|908402612|probable glutathione S-transferase 7 [Biomphalaria glabrata]	1	2	18	24.455	23679333	
25	>c260447_g1	alanine--glyoxylate aminotransferase 2, mitochondrial-like [Aplysia californica]	1	4	13.5	32.278	1.31E+08	
26	>c260469_g1	gi|524886899|EF-hand calcium-binding domain-containing protein 6-like [Aplysia californica]	1	10	8	180.33	32195667	
27	>c263520_g1	gi|524866607|BTB/POZ domain-containing protein 19-like [Aplysia californica]	1	5	21.8	34.343	42835333	
28	>c266112_g1	gi|919002296|cilia- and flagella-associated protein 44-like [Lingula anatina]	1	3	7	63.166	24476000	
28	>c266550_g5	gi|871248298|EF-hand calcium-binding domain-containing protein 5-like [Aplysia californica]	1	4	9.9	59.797	23932333	
30	>c267093_g5	radial spoke head 1 homolog [Aplysia californica]	1	1	31.8	9.7533	23769000	
31	>c267924_g1	gi|871270068|intraflagellar transport protein 56-like [Aplysia californica]	1	2	5.6	64.285	18620000	
32	>c335024_g1	gi|908450794|rootletin-like [Biomphalaria glabrata]	1	3	36.5	14.482	45410667	
33	>c215021_g1	gi|221107099|cathepsin L1-like, partial [Hydra vulgaris]	1	1	39.5	4.2498		30267333
	>c223366_g1	gi|961899123|C-type lectin domain family 10 member A-like	1	1	9.3	14.233		21688000

34		[Poecilia mexicana]					
35	>c235706_g1	gi|1000738555|histone H2B-like [Cephus cinctus]	1	1	25.6	13.984	3.56E+08
36	>c257851_g1	rootletin-like [Biomphalaria glabrata]	1	3	11.6	42.54	20865667
37	>c265793_g4	gi|312190476|serine protease inhibitor protein [Haliotis discus discus]	1	2	13.8	17.93	17203667
38	>c266065_g1	gi|1031915608|cathepsin L [Meretrix meretrix]	1	1	39.5	4.3178	88765000
39	>c267550_g1	gi|871230524|xaa-Pro aminopeptidase 1-like [Aplysia californica]	1	1	1.8	61.762	18274000
40	>c277191_g1; >c165872_g1; >c225664_g2	gi|1007753279|heat shock cognate 70 kDa protein [Nothobranchius furzeri]	3	1	25	40.412	81343667
41	>c295622_g1	gi|121634|Glycine-rich protein GRP33 [Artemia salina]	1	1	22.5	4.4112	29910667
42	>c368924_g1	gi|724970559|heat shock 70 kDa protein cognate 4-like, partial [Rhinopithecus roxellana]	1	2	50	9.866	49336667

Supplementary Table 4 Primer sequences for realtime PCR assay

No	Designated name		Primers
1	actin	F	TCTTGGGTATGGAATCTGCTGG
		R	CCTTTTGCATTCTGTCAGCGAT
2	EF1-F	F	GTTCGAGAAAGAAGCCCAGGA
		R	GTCTCGAACTTCCACAGAGCA
3	chymotrypsin	F	GAACTCCTGCAAACAACACTGG
		R	TCATCTTGTTGGTCCTGTGGTC
4	Amylase	F	GGACTTCCTCAACCACCTCATT
		R	GACTCTTCCAGACTGCAGGTAC
5	Cellulase	F	CGTTGAACTCGGTCACCCATA
		R	GGTCGTCAAGCTCTTCCTCAA
6	EF-hand calcium-binding domain-containing protein	F	AATCGACAGAAGAAGACCCAGAC
		R	GCCTCTTTGAAGTCTGAGAAGGA
7	calcium-binding mitochondrial carrier protein SCaMC	F	CGGATCATGTACATCTTCTTCAGC
		R	CTTTCTACAGAGGCTACATCCCAA
8	Calmodulin	F	GAATTCACACACCTGTACACACTG
		R	CTGTAGGGTCATCAATTTTGGCTC
9	C-type lectin	F	TGGGCTGATGGAATGATAGTGTG
		R	CCGAGCCATATCTCTTCAGGTAA
10	Lysozyme	F	GTTCAGCTCATGGTAATCGTTGC
		R	GCTACAACTGTTTCGGTGACTTC
11	stress protein A	F	CCTTTCCTCCAATCCCATGAGAT
		R	GTGACCATGTGATACTGTTGCAC
12	glutathione s-transferase	F	GAAGACCGAGAAAGAAAAGTCGC
		R	GAACACTTCGTCCACTCTCAATG
13	Hsp70	F	CAATGGTATCCTCAATGTGTCTGC
		R	CTTCTCATCCTCCTCCTTGAACTTG
14	cathepsin L	F	GAGAATTGTCACTTTTCCCCTGC
		R	GTAGTAGACTCCGGACCTGTAGAG
15	14-3-3	F	CTGGAGCCGATGGTATTCTTGTA
		R	GGCTAAGATCACCGAACAAACTG
16	SCO-spondin	F	GAAATCGTGTGAATGCTACCCG
		R	CTGAACATTCCGTCCAGTCTGA
17	kyphoscoliosis peptidase	F	GTCAGGTGGAGAAGGAACGATT
		R	GTGTTGCGTAGTTGGTCTTGTC
18	radial spoke head 1 homolog	F	CCTCCATTCGGTTCTCCTTGAA
		R	GGTTCGGCAAGTACTACTACGT
19	marginal zone B- and B1-cell-specific protein-like	F	GCTTCATCCTTTGTTTCTAGCCC
		R	CATGCCATCAGATCTTATGTGCG
